# A systematic review of the safety and efficacy of artemether-lumefantrine against uncomplicated *Plasmodium falciparum* malaria during pregnancy

**DOI:** 10.1186/1475-2875-11-141

**Published:** 2012-05-01

**Authors:** Christine Manyando, Kassoum Kayentao, Umberto D’Alessandro, Henrietta U Okafor, Elizabeth Juma, Kamal Hamed

**Affiliations:** 1 Tropical Diseases Research Centre, Ndola, Zambia; 2 Malaria Research and Training Centre, Bamako, Mali; 3 Institute of Tropical Medicine, Antwerp, Belgium; 4 Medical Research Council Unit, Fajara, The Gambia; 5 Department of Paediatrics, College of Medicine, University of Nigeria, Enugu, Nigeria; 6 Kenya Medical Research Institute, Kisumu, Kenya; 7 Novartis Pharmaceuticals Corporation, East Hanover, NJ, USA

**Keywords:** Artemether-lumefantrine, Artemisinin-based combination therapy (ACT), Pregnancy, Malaria, *Plasmodium falciparum*

## Abstract

Malaria during pregnancy, particularly *Plasmodium falciparum* malaria, has been linked to increased morbidity and mortality, which must be reduced by both preventive measures and effective case management. The World Health Organization (WHO) recommends artemisinin-based combination therapy (ACT) to treat uncomplicated *falciparum* malaria during the second and third trimesters of pregnancy, and quinine plus clindamycin during the first trimester. However, the national policies of many African countries currently recommend quinine throughout pregnancy. Therefore, the aim of this article is to provide a summary of the available data on the safety and efficacy of artemether-lumefantrine (AL) in pregnancy. An English-language search identified 16 publications from 1989 to October 2011 with reports of artemether or AL exposure in pregnancy, including randomized clinical trials, observational studies and systematic reviews. Overall, there were 1,103 reports of AL use in pregnant women: 890 second/third trimester exposures; 212 first trimester exposures; and one case where the trimester of exposure was not reported. In the second and third trimesters, AL was not associated with increased adverse pregnancy outcomes as compared with quinine or sulphadoxine-pyrimethamine, showed improved tolerability relative to quinine, and its efficacy was non-inferior to quinine. There is evidence to suggest that the pharmacokinetics of anti-malarial drugs may change in pregnancy, although the impact on efficacy and safety needs to be studied further, especially since the majority of studies report high cure rates and adequate tolerability. As there are fewer reports of AL safety in the first trimester, additional data are required to assess the potential to use AL in the first trimester. Though the available safety and efficacy data support the use of AL in the second and third trimesters, there is still a need for further information. These findings reinforce the WHO recommendation to treat uncomplicated *falciparum* malaria with quinine plus clindamycin in early pregnancy and ACT in later pregnancy.

## Background

As pregnant women are at increased risk of malaria it is essential to ensure that preventive measures, accurate diagnosis and effective treatments are accessible to this vulnerable group [1]. Although all *Plasmodium* species can affect maternal and foetal health, *Plasmodium falciparum* infection is associated with the most severe effects and has been linked to increased maternal, foetal and neonatal morbidity and mortality [1-3]. In 2007, an estimated 85.3 million pregnancies occurred in regions of *P. falciparum* transmission worldwide. Of these, the majority occurred in the African (30 million), South East Asian and Western Pacific (44.2 million) regions. Within the African region, 29.6 million pregnancies occurred in areas of stable transmission and 0.4 million pregnancies occurred in areas of unstable transmission [4]. In sub-Saharan Africa, where both malaria and HIV are prevalent, the population-attributable fraction of malaria cases due to HIV during pregnancy was estimated at 4.8%, corresponding to an additional 505,382 pregnant women with malaria each year [5].

In regions of stable malaria transmission, frequent exposure to malaria parasites leads to the development of partial immunity, and malaria infection is often asymptomatic or without specific symptoms. Nevertheless, malaria infection is associated with severe maternal anaemia, placental malaria and low birth weight – a risk factor for infant death [6,7]. Endemic malaria has been associated with increased perinatal and foetal mortality, risk of abortion, and premature delivery [1,2]. In areas of unstable transmission where adults are unlikely to have acquired immunity against malaria, pregnant women are at increased risk of severe malaria, which can cause maternal and foetal death [1,6]. In a hospital study in Mozambique, maternal death from severe malaria was more frequent in urban (12.5%) and suburban (14.3%) areas where transmission was low as compared to rural areas with moderate or stable transmission (3.6%) [3]. In a large study of 1,030 pregnant women in Mozambique, acute placental infection and parasitaemia in cord blood were significantly associated with infant death, while maternal clinical *falciparum* malaria and acute placental infection were associated with clinical malaria during infancy [8].

The World Health Organization (WHO) recommends the use of preventive measures to limit the occurrence of malaria in pregnancy, including the use of insecticide-treated nets and intermittent preventive treatment in pregnancy (IPTp) in areas of stable malaria transmission. The WHO also recommends that all cases of malaria during pregnancy should be treated promptly with an effective anti-malarial [6,9].

## ACT in pregnancy

Artemisinin-based combination therapy (ACT) is effective and well tolerated in the general population and, if shown to be safe and effective, may significantly reduce morbidity and mortality during pregnancy [10]. Hence, following 1,500 documented pregnancies exposed to artemisinin derivatives during the second or third trimester, the WHO assessed the benefits and potential risks and recommended the use of ACT as a first-line treatment for uncomplicated *P. falciparum* malaria during the second and third trimesters [11]. Those recommended are artemether-lumefantrine (AL), artesunate-amodiaquine (AS-AQ), artesunate-mefloquine (AS-MQ) and artesunate + sulphadoxine-pyrimethamine (AS + SP), but do not include dihydroartemisinin-piperaquine (DHA-PPQ) due to the lack of data in the second and third trimesters in 2010, when the latest edition of the WHO guidelines was published [11].

ACT is not recommended by the WHO as a first-line treatment during the first trimester, due to limited clinical safety data [11] and evidence of embryo lethality and developmental abnormalities in animal studies following artemisinin exposure early in pregnancy, at times equivalent to the first trimester in humans [12,13]. The WHO recommends a seven-day course of quinine plus clindamycin (or quinine monotherapy if clindamycin is not available) as the first-line malaria treatment during the first trimester of pregnancy, and artesunate plus clindamycin as second-line treatment. ACT in the first trimester of pregnancy is indicated only if the recommended treatments are not available or have failed [11].

It is vital that pregnant women with malaria receive prompt and effective treatment [11]. However, the frequent exclusion of pregnant women from pharmaceutical trials, including the original studies of artemisinins and different types of ACT, has limited the availability of data in this vulnerable population. Consequently, ACT safety and efficacy, including potential effects of changes in ACT pharmacokinetics during pregnancy, is an important area of malaria research with implications for national malaria control policies. An understanding of the risks and benefits of ACT during early and later pregnancy is needed to guide policymakers and health-care workers, and ensure that pregnant women receive both effective and safe treatment against malaria.

The aim of this review is to discuss the available data on the maternal and foetal safety, pharmacokinetics, and anti-malarial efficacy of AL during pregnancy. As the first fixed-dose ACT, AL has now been available for over ten years and has been widely used as a first-line anti-malarial in many countries worldwide, including sub-Saharan Africa. Hence, there is a growing body of data on the safety and efficacy of AL in pregnant women.

## Methods

A literature search was performed to identify publications that included specific reports of artemether or AL exposure during human pregnancy and any safety, efficacy or pharmacokinetic outcome. This included original research studies and systematic reviews published in any year. The search was limited to English-language publications. Ovid was used to search EMBASE, Medline, Biosis and the Cochrane database (16 December, 2011) according to the search strategy shown in Additional file 1. The Malaria in Pregnancy consortium library was searched using the following sets of search terms (8 December, 2011): artemether AND pregnancy; artemether-lumefantrine AND pregnancy; lumefantrine AND pregnancy; artemisinins AND pregnancy; ACT AND pregnancy AND malaria; ACT AND pregnancy AND *Plasmodium falciparum.* The same terms were used to search Clinicaltrials.gov (8 December, 2011) and the WHO International Clinical Trials Registry Platform (ICTRP) (8 December, 2011) to identify any publications related to the identified clinical trials, and TrialTrove 5 was searched (14 December, 2011) between 2008 and 2011 using the following search combinations: artemether + lumefantrine OR artemether OR artemether + lumefantrine Cipla (Cipla is a manufacturer of AL) OR lumefantrine OR artemisinins AND pregnancy OR pregnant; artemether + lumefantrine OR artemether OR artemether + lumefantrine Cipla AND pregnancy OR pregnant AND malaria OR *Plasmodium falciparum*. References of identified manuscripts were searched for additional reports.

The manuscripts identified in the search were screened manually based on their titles and (where available) abstracts and if appropriate on the full text to identify those that included reports of artemether or AL exposure and any safety, efficacy, or pharmacokinetic outcomes in human pregnancy. Previous systematic reviews that included artemether in human pregnancy were also selected. As each article used separate outcome measures, the safety (maternal, foetal, and infant), efficacy and pharmacokinetic outcomes of each report were recorded and included in the review. The type of study, location, dates, and relevant study protocols were also included to put the data in context and to highlight limitations. The data from each study were considered separately.

## Results

**Figure 1:**
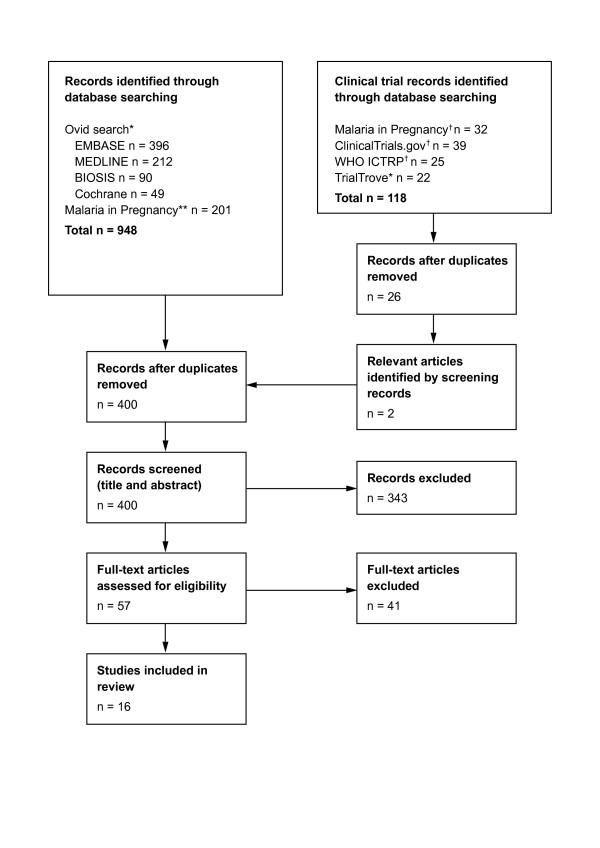
**Flow diagram showing the number of records retrieved, screened and included in this review article.** Records were identified by searching publication and clinical trial databases. Duplicates were removed and the remaining records were screened based on their titles, abstracts or clinical trial information. Based on this screen, records judged not to feature any data on the subject of artemether or AL safety, efficacy or pharmacokinetics in human pregnancy were excluded. Of 57 full text articles assessed for eligibility, 39 articles were excluded as they did not include specific reports of artemether or AL exposures during pregnancy and two articles were excluded to avoid duplication of pregnancy exposures. Sixteen relevant articles were therefore identified and included in this review article *1948–2011; **English-language articles. All years; ^†^All years; ICTRP, International Clinical Trials Registry Platform.

The database searches identified 948 records from the publication search and 118 clinical trial records ( Additional file 2). After removal of duplicates, 400 records were screened based on their titles and (where available) abstracts (Additional file 3). Of these, 343 were judged not to feature any data on the subject of artemether or AL safety, efficacy or pharmacokinetics in human pregnancy and were excluded. Of 57 full text articles assessed for eligibility, 39 articles were excluded as they did not include specific reports of artemether or AL exposures during pregnancy and two articles were excluded to avoid duplication of pregnancy exposures. Sixteen relevant articles were identified (Figure 1). These were published between 1989 and October 2011, and included specific reports of artemether or AL exposure in pregnancy in either randomized clinical trials or observational studies and also previous systematic reviews ( Additional file 4).

In total, these articles contain reports of 1,236 artemether exposures during pregnancy, including 964 in the second and third trimesters, 261 exposures in the first trimester, and 11 where the timing of exposure was not reported. Of these, 1,103 related to AL exposures, with 890 in the second and third trimesters, 212 in the first trimester, and one case where the timing of exposure was not reported ( Additional file 4).

## Studies assessing the safety of artemether and AL during pregnancy

The safety of artemisinins in pregnancy has been investigated in the second and third trimesters in randomized clinical trials and throughout pregnancy in observational studies. A 2007 review of all artemisinin derivatives used in pregnancy identified 14 studies, reporting a total of 945 pregnancies exposed to artemisinins (artesunate or artemether): 123 in the first trimester and 822 in the second and third trimesters. Five of these studies were randomized clinical trials; nine were descriptive non-randomized studies. Women were followed up to delivery, and 214 infants were examined up to at least one year of age. Overall, these studies did not identify any effect of artemisinins on serious maternal adverse events, adverse birth outcomes or neurological development deficits in the infants [14].

Within the review by Dellicour *et al.*[14], a total of 86 pregnancies were exposed to artemether across four studies: one in Nigeria, one in Sudan, one in Thailand, and one in China (Tables 1 and 2, Additional file 4). In the Nigerian study, 45 pregnant women with drug-resistant *falciparum* malaria were treated with artemether alone or artemether then mefloquine between 1994 and 1997. The treatments were all administered in the second or third trimester of pregnancy. All infants were normal at birth and showed normal physical and neurological development for the period of assessment, which varied between six and 36 months [15]. The study in Sudan was conducted between 1997 and 2001, and a total of 28 pregnant women with chloroquine- and quinine-resistant *falciparum* malaria were treated with intramuscular artemether. Only one woman received artemether treatment during the first trimester. In this study, there were no maternal deaths, miscarriages, stillbirths or reports of congenital abnormalities, but there was one perinatal death following a pre-term labour [16]. In the Thai study, 11 pregnant women with acute *falciparum* malaria received artemether, either alone or combined with mefloquine, artesunate, clindamycin, or lumefantrine between 1992 and 2000 [17]. The paper by McGready *et al.*[17] includes all of the artemether exposures reported across three publications. To avoid duplication of pregnancy exposures, the other two reports have not been included [18,19]. Nine women were available for follow-up, and of these, seven delivered normal infants. There was one miscarriage and one maternal death from severe malaria and anaemia [17]. In the Chinese study, two pregnant women with typical signs and symptoms of malaria were treated with artemether in 1980. Both women were in the second trimester of pregnancy, both delivered at full term and both children showed normal growth and development at five years [20].

**Table 1:** Maternal and infant safety outcomes of artemether and lumefantrine use during pregnancy

**Publication**	**Drug (n)**	**Trimester**		**Maternal outcomes**			**Infant outcomes**	
		**1**	**2/3**	**AE**	**SAE**	**Death**	**Normal development**	**Death, 1 yr**
**Wang 1989**[20]	A	0	2				2/2^a^	
**Sowunmi*et al.,*1998**[15]	A + MQ (22), A (23)	0	45	Minimal			Yes^b^	
**McGready*et al.*, 2001**[17]	A + MQ/AS/C (10), AL (1)	–	–		0/9^c^	1/9^d^		
**Adam*et al.,*2004**[16]	A	1	27			0/28		
**McGready*et al.,*2006**[21]	AL	0	13*		0/13		9/10^e^	
**McGready*et al.*, 2008**[22]	AL	0	125		1^c,f^	1^g^		1/117 (0.9%)^h^
	AS	0	128		0^c^	0		8/120 (6.7%)^i^
**Kaye*et al.*, 2008**[23]	AL	0	58	7				
**Adam*et al.,*2009**[24]	A (48), AL (3)	51	0			0		0/49
**Piola*et al.*, 2010**[25]	AL	0	152	94/152 (61.8%)^j^		0		
	Q	0	152	142/152 (93%)^j^		1^k^		
**Manyando*et al.*, 2010**[26]	AL^l^	156	348		108/495 (21.8%)	1 (0.2%)^m^		
	SP/Q^l^	138	378		SP 118/506 (23.3%)	5 (1%)^n^		
**Sangaré*et al.,*2011**[27]	AL (260)^o^	53^p^	207					

*A* artemether; *AE* adverse events; *AL* artemether-lumefantrine; *AS* artesunate; *C* clindamycin; *IM* intramuscular; *MQ* mefloquine; *Q* quinine; *SAE* serious adverse event; *SP* sulphadoxine-pyrimethamine; *These 13 exposures are not included in the total count of exposures to AL in pregnant women as they were also included in McGready, *et al.* 2008 [22]; ^a^5 years; ^b^physical and neurodevelopment within normal limits (6–36 months); ^c^drug related; ^d^severe malaria and anaemia; ^e^1 year; ^f^Day 2 increase in parasitaemia; ^g^haemorrhagic shock (treatment not suspected); ^h^suspected intraventricular haemorrhage, background of Allagile’s syndrome; ^i^3 related to prematurity, 2 pneumonia, 1 sepsis, 1 diarrhoea, 1 suspected beriberi; ^j^number reporting at least 1 AE; ^k^sepsis following a caesarean section; ^l^AL group n = 495, AL exposure n = 504, SP group n = 506, SP/Q exposure n = 516; ^m^anaemia secondary to spontaneous abortion; ^n^1 related to Kaposi’s sarcoma, 3 infections, 1 undiagnosed illness; ^o^260 reports of AL treatment for an episode of malaria; ^p^26 received only AL in the 1st trimester.

**Table 2:** Pregnancy outcomes of artemether and lumefantrine use during pregnancy

**Publication**	**Drug (n)**	**Trimester**		**Pregnancy outcomes**					
		**1**	**2/3**	**Prematurity**	**Miscarriage**	**Stillbirth**	**Congenital abnormality**	**Low birth weight**	**Neo-natal death**
**Wang 1989**[20]	A	0	2	0/2			0/2^a^		
**Sowunmi*et al.,*1998**[15]	A + MQ (22), A (23)	0	45						
**McGready*et al.*, 2001**[17]	A + MQ/AS/C (10), AL (1)	–	–		1/9				
**Adam*et al.,*2004**[16]	A	1	27	1/28^b^			0/28		
**McGready*et al.,*2006**[21]	AL	0	13*	1^c^			0		
**McGready*et al.*, 2008**[22]	AL	0	125	3/117 (2.6%)	0/125	1/125 (0.8%)	3/117 (2.6%)	14/99 (14.1%)	
	AS	0	128	10/120 (8.3%)	1/128 (0.8%)	1/128 (0.8%)	4/120 (3.3%)	20/101 (19.8%)	
**Kaye*et al.*, 2008**[23]	AL	0	58						
**Adam*et al.,*2009**[24]	A (48), AL (3)	51	0		2^d^		0		
**Piola*et al.*, 2010**[25]	AL	0	152	12/143 (8.4%)	3/144 (2.1%)	2/144 (1.4%)	3/143 (2.1%)	12/120 (10.2%)	3/144 (2.1%)
	Q	0	152	17/137 (12.4%)	4/137 (2.9%)	3/137 (2.2%)	2/137 (1.5%)	16/119 (13.4%)	6/137 (4.4%)
**Manyando*et al.*, 2010**[26]	AL^e^	156	348	71/504 (14.1%)	7/504 (1.4%)	9/504 (1.8%)	8/449 (1.8%) (29/449 [6.5%])^f^	9.0%	11/475 (2.3%)
	AL 1^st^ trimester	156		20/150 (13.3%)	7/159 (4.4%)^g^	2/135 (1.5%)	1/130 (0.8%) (9/130 [6.9%])^f^		4/135 (3%)
	SP/Q^e^	138	378	90/516 (17.4%)	8/516 (1.6%)	13/516 (2.5%)	SP 6/444 (1.4%) (18/444 [4.1%])^f^	7.7%	11/480 (2.3%)
	SP/Q 1^st^ trimester	138		28/135 (20.7%)	0/135	3/129 (2.3%)	3/121 (2.5%) (8/121 [6.6%])^f^		2/129 (1.6%)
**Sangaré*et al.,*2011**[27]	AL (260)^h^	53^i^	207			7/500^j^			

*A* artemether; *AL* artemether-lumefantrine; *AS* artesunate; *C* clindamycin; *MQ* mefloquine; *Q* quinine; *SP* sulphadoxine-pyrimethamine; *These 13 exposures are not included in the total count of exposures to AL in pregnant women as they were also included in McGready *et al.*, 2008 [22]; ^a^5 years; ^b^infant died, 2^nd^–3^rd^ trimester exposure; ^c^associated with maternal urinary tract infection; ^d^A group, associated with quinine infusion for further malaria attack; ^e^AL group n = 495, AL exposure 504, SP group n = 506, SP/Q exposure n = 516; ^f^including umbilical hernia; ^g^2 multiple malaria episodes, 1 syphilis, 1 concomitant salbutamol and AL for threatened abortion; ^h^260 reports of AL treatment for an episode of malaria; ^i^26 received only AL in the 1st trimester; ^j^total study population.

Since the Dellicour review, seven studies have reported on the safety of AL during pregnancy: four observational studies and three open-label randomized studies (Tables 1 and 2, Additional file 4). These studies reported on the tolerability of AL during pregnancy and the pregnancy outcomes.

A large prospective cohort study comparing the safety of AL and sulphadoxine-pyrimethamine (SP) throughout pregnancy was recently conducted in Zambia, where SP was the recommended treatment for uncomplicated *falciparum* malaria in the second and third trimesters of pregnancy at that time [26]. In total, 495 women had received AL and 506 had received SP to treat the index episode of malaria. Diagnosis was predominantly based on clinical symptoms, and was unconfirmed by microscopy or rapid diagnostic test (RDT) in 85% of cases. As women in Zambia were recommended to receive SP as IPTp in the second and third trimesters, 412 women in the AL group had also received SP, while only 13 women in the SP group had also received AL. There were no significant overall differences in the rates of maternal mortality (AL, 0.2%; SP, 1.0%), neonatal mortality (AL, 2.3%; SP, 2.3%), stillbirth (AL, 1.8%; SP, 2.5%), miscarriage (AL, 1.4%; SP, 1.6%), low birth weight (AL, 9.0%; SP, 7.7%), birth defects (AL, 1.8%; SP, 1.4% [including umbilical hernia: AL, 6.5%; SP, 4.1%]) and infant neurological development between the AL and SP groups. Premature delivery was slightly more frequent in the SP group than the AL group (17.4% *versus* 14.1%, respectively) [26].

The Zambian study also assessed the pregnancy outcomes according to the timing of the anti-malarial treatment. Within the population, 156 women received AL in the first trimester and 138 women received SP and/or quinine in the first trimester. The rates of perinatal deaths (stillbirths and neonatal deaths) were similar for women receiving AL or SP in the first trimester (4.4% AL, 3.9% SP and/or quinine), as were the rates of infant malformations (0.8% AL, 2.5% SP and/or quinine [including umbilical hernia: AL, 6.9%; SP, 6.6%]) and assessments of infant neurodevelopment. However, there were seven miscarriages, four with confounding factors (two women had multiple malaria episodes, one had syphilis and one received AL and concomitant salbutamol for a threatened miscarriage), in women exposed to AL in the first trimester, and none in the SP group. As a result, the authors recommended further monitoring of ACT use in the first trimester [26].

Another recent observational study investigated the safety of artemisinins in the first trimester of pregnancy in Sudan between 2006 and 2008 [24]. In total, 62 women had received artemisinins in the first trimester: 48 received artemether injections, 11 received AS + SP, and three received AL. Within this study population, there were no maternal deaths and 60 infants were born at full term. No congenital malformations were reported and all 60 infants survived to the final one-year time-point. Two women who had received artemether injections in the first trimester miscarried while receiving a quinine infusion for a subsequent malaria attack (20 weeks and 22 weeks) [24].

A study of the pharmacokinetics of AL during pregnancy, conducted in Thailand in 2004, also reported on the safety of AL [21]. In total, 13 women in the second and third trimesters of pregnancy received AL to treat recrudescent *falciparum* malaria after seven days of supervised quinine treatment. All were rapidly cured of their infection (median parasite clearance time was two days). There were no reports of serious adverse events and all 13 women delivered live infants with no congenital abnormalities. Ten infants were available for follow-up at one year and of these, nine showed normal development and one showed severe developmental delay [21].

The fourth observational study was a retrospective survey of anti-malarial use for self-reported episodes of malaria conducted in Uganda between 2008 and 2009 [27]. Women with a pregnancy in the previous 12 months that reached at least the third trimester were included in the study, while current pregnancies were excluded. These inclusion criteria prevent the assessment of the occurrence of miscarriages prior to the third trimester. Of 500 women, 334 reported 637 episodes of malaria in their last pregnancy. In the first trimester, 53 of 134 malaria episodes were treated with AL alone (26) or AL and other anti-malarials (27). In the second and third trimesters, 207 of 503 malaria episodes were treated with AL alone (113) or AL and other anti-malarials (94). Safety outcomes were not reported by drug or exposure time, but over the 500 pregnancies there were seven stillbirths (1.4%) and 493 live births (98.6%) [27].

The safety of AL and quinine was recently compared in an open-label randomized non-inferiority trial in Uganda [25]. In total, 304 women in the second or third trimester of pregnancy with uncomplicated *falciparum* malaria were treated with either a three-day course of AL (152) or a seven-day course of quinine (152). No significant differences were evident between the groups in the frequency of miscarriages (AL, 2.1%; quinine, 2.9%), stillbirths (AL, 1.4%; quinine, 2.2%), early neonatal deaths (AL, 2.1%; quinine 4.4%), malformations (AL, 2.1%; quinine, 1.5%), prematurity (AL, 8.4%; quinine, 12.4%) and low birth weight (AL, 10.2%; quinine 13.4%). Laboratory tests also showed no clinically significant adverse events and no substantial differences in alanine aminotransferase, bilirubin, creatinine, and full blood count between the groups. However, in terms of maternal tolerability, fewer women in the AL group than the quinine group reported at least one adverse event (61.8% *versus* 93%, respectively). Tinnitus, nausea, vomiting and anorexia were the most commonly reported events in the quinine group, with abdominal pain, headaches and influenza-like syndrome most common in the AL group. There was also one maternal death in the quinine group from sepsis following a caesarean section [25].

The safety of AL and artesunate in the second and third trimesters of pregnancy were compared in an open-label randomized study in Thailand between 2004 and 2006 [22]. Women with acute *falciparum* malaria were randomized to treatment: 125 to AL and 128 to artesunate (seven days). Birth outcomes were assessed and were similar between the two treatment groups. There was one reported miscarriage in the artesunate group and one stillbirth in each treatment group. No significant differences were detected between the treatment groups in the occurrence of congenital abnormalities (AL, 2.6%; artesunate, 3.3%), prematurity (AL, 2.6%; artesunate, 8.3%) and low birth weight (AL, 14.1%; artesunate, 19.8%). The authors reported that there were no differences between the groups in terms of growth or developmental parameters at one year after birth, but that there were significantly more deaths in the artesunate group (6.7%) than the AL group (0.9%), although these deaths were unlikely to be related to treatment. Tolerability of the two treatments was similar, with only one serious adverse event related to treatment (Day 2 increase in parasitaemia in the AL group) and one maternal death, which was not suspected to be related to the AL treatment (haemorrhagic shock after a ruptured uterus). Haematology and blood chemistry evaluations detected no difference in the occurrence of abnormal values at Day 14 between the treatment groups, and paired ECG examinations (baseline and 1 hour after treatment) showed no clinically relevant abnormalities after AL treatment. The only adverse event that differed between the treatment groups was tinnitus, which was more frequent in the artesunate group (AL, 0%; artesunate, 8.5%) [22].

A 2006 Ugandan open-label, randomized study compared AL with chlorproguanil-dapsone in the second and third trimesters of pregnancy for the treatment of uncomplicated *falciparum* malaria [23]. Chlorproguanil-dapsone has now been withdrawn, and so only the results from the AL arm of the study will be discussed [28]. This study reported the occurrence of maternal adverse events, but did not report on pregnancy outcomes. In total, 58 women were randomly assigned to AL treatment. Liver and renal function tests remained normal throughout the study and the frequency of adverse events was low (palpitations (4), dizziness (1), drowsiness (1) and generalized skin rash (1)) [23].

## Studies assessing the efficacy of AL during pregnancy

The efficacy of AL against uncomplicated *falciparum* malaria during the second and third trimesters of pregnancy was investigated in the three open-label randomized studies described above (Table 3Additional file 4). In the Ugandan study that compared AL with quinine, 152 women with positive blood smears were randomized to each treatment. This study reported high efficacy of AL during pregnancy and the non-inferiority of AL relative to quinine. The PCR-corrected cure rates were similar both at Day 42 (AL, 99.3% (range 96.0–99.9); quinine, 97.6% (range 93.1–99.5)) and at delivery (or Day 42 if later) (AL, 98.2% (range 93.5–99.7); quinine, 96.1% (range 90.2–98.9)) [25]. The Ugandan study of AL *versus* chlorproguanil-dapsone, where 58 pregnant women with clinical symptoms of malaria and positive blood smears were randomized to AL, also reported high efficacy of AL with a 28-day cure rate of 100% for both treatments [23]. However, the Thai study comparing AL and artesunate treatment in women with positive blood smears (AL, 125 women; AS, 128 women) reported a cure rate of less than 95% for AL at Day 42 and for both treatments at delivery (or Day 42 if later). In this study, both treatment groups had a median (range) time to parasite clearance of 2 (1–5) days, but the PCR-adjusted cure rate at Day 42 was 95.2% (95% CI 91.5–97.1) for artesunate and 87.2% (95% CI 81.1–93.2) for AL, and at delivery (or Day 42 if later) was 89.2% (95% CI 82.3–96.1) for artesunate and 82.0% (95% CI 74.8–89.3) for AL. Before delivery (or Day 42 if later), there were 20 recrudescent infections with AL and 10 with artesunate [22].

**Table 3:** Efficacy outcomes of artemether–lumefantrine use during pregnancy

**Publication**	**Description**	**Treatment (n)**	**Fever clearance**	**Parasite clearance**	**Cure rate**
**McGready*et al.*, 2008**[22]	Thai study. Open- label, randomized study of AL *vs.* AS in the 2^nd^ and 3^rd^ trimesters	AL (125)	2 (1–3) days^†^	2 (1–5) days^†^	87.2% (95% CI 81.1–93.2)^‡^ at Day 42.82.0% (95% CI 74.8–89.3)^‡^ at delivery (or Day 42 if later)
		AS (128)	1 (1–2) days^†^	2 (1–5) days^†^	95.2% (95% CI 91.5–97.1)^‡^ at Day 42.89.2% (95% CI 82.3–96.1)^‡^ at delivery (or Day 42 if later)
**Kaye*et al.*, 2008**[23]	Ugandan study. Open-label, randomized study of AL *vs.* chlorproguanil-dapsone* in the 2^nd^ and 3^rd^ trimesters	AL (58)	47 (85.5%) clear at Day 2	49 (89.1%) clear at Day 2	100% at Day 28
**Piola*et al.,*2010**[25]	Ugandan study. Open-label, randomized study of AL *vs.* Q in the 2^nd^ and 3^rd^ trimesters	AL (152)	130 (100%) clear at Day 2	148 (99%) clear at Day 2	99.3% (range 96.0–99.9)^§^ at Day 42.98.2% (range 93.5–99.7)^§^ at delivery (or Day 42 if later)
		Q (152)	127 (99%) clear at Day 2	123 (86%) clear at Day 2	97.6% (range 93.1–99.5)^§^ at Day 42.96.1% (range 90.2–98.9)^§^ at delivery (or Day 42 if later)

*AL* artemether-lumefantrine; *AS* artesunate; *CI* confidence interval; *Q* quinine; *Chlorproguanil-dapsone has been withdrawn, so the chloroproguanil-dapsone data are not shown; ^†^median (range) days; ^‡^PCR-adjusted, intent-to-treat population; ^§^PCR-adjusted, modified intent-to-treat population.

## Studies assessing the pharmacokinetics of AL during pregnancy

It has been reported that the pharmacokinetics of anti-malarial treatments may change during pregnancy [29,30]. There are several reports of the pharmacokinetics of AL during pregnancy, but there are currently no published data directly comparing the pharmacokinetics in pregnant and non-pregnant women (Table 4). A study conducted in Thailand in 2004 assessed the lumefantrine and artemether exposure in a small number (13) of women in the second or third trimester of pregnancy, who were still infected after seven days of supervised quinine treatment. Results were compared with those of a previous report from a small cohort (17) of non-pregnant adult malaria patients [21]. All 13 pregnant women were cured rapidly, with parasite clearance within one to three days. Although data sets were relatively limited by the small number of patients, lumefantrine plasma concentration-time curves for pregnant and non-pregnant patients were very similar within the first five days after treatment (i.e. within the relevant therapeutic window). Lumefantrine total exposure (Area Under the Curve [AUC]) from 60 hours after treatment initiation (after last dose) until time infinity (AUC_60h→∞_) was 237 μg/ml.h (90% predicted range 75–576) in pregnant women *versus* 251 μg/ml.h (90% predicted range 79–616) in non-pregnant adults, thus differing overall by only 6% [21,31]. In addition, the median lumefantrine C_max_ of 7.34 μg/ml reported in pregnant women in this study was well within the range of values reported in non-pregnant female and male patients in several other studies [31-33]. Artemether exposure was also described as being reduced in this pregnant population study compared with previous reports from non-pregnant adults (pregnant women, median AUC_0→8h_ of 66.4 ng/ml.h (90% range 10.5–264.8); non-pregnant adults, mean AUC_0→8h_ 211 ng/ml.h (SD 109)) [21]. This comparison was made with exposure values from 25 male Thai malaria patients [34]. However, other reports in non-pregnant individuals also given the six-dose regimen of AL [35,36] showed comparable or even lower artemether and dihydroartemisinin (DHA) exposure values than those reported in pregnant women in the study by McGready *et al.*, [21]. These exposure values were measured in healthy subjects, who are known to exhibit the highest artemether and lumefantrine absorption and bioavailability when the drug is given with a standardized meal. Therefore, and because of the considerably large inter-subject (and inter-study) variability in the pharmacokinetics of artemether and lumefantrine, comparisons with historical data should be done with great caution. Furthermore, despite apparently lower exposure to artemether and DHA in the study by McGready *et al.*, [21] all 13 pregnant women treated with AL were cured. In one woman, *P. falciparum* infection reappeared during follow-up (Day 21), but PCR genotyping confirmed this to be a new infection.

**Table 4:** Pharmacokinetics of artemether–lumefantrine during pregnancy

**Publication**	**Description**	**Median (range) Day 7 lumefantrine concentration**	**Median (range) lumefantrine C**_**max**_	**Lumefantrine exposure**	**Artemether exposure (AUC**_**0→8h**_**)**
**McGready*et al.,*2006**[21]	Pharmacokinetic study of AL in pregnancy using venous plasma samples (n = 13). Results compared with a previous report in non-pregnant adults (n = 17)	384 ng/ml (62–835)*	7340 ng/ml (1,590–15,670)*	Pregnant: 237 μg/ml.h (90% predicted range 75–576)^†^, Non-pregnant: 251 μg/ml.h (90% predicted range 79–616)^†^	Pregnant, median (90% range): 66.4 ng/ml.h (10.5–264.8), Non-pregnant, mean (SD): 211 ng/ml.h (109)
**Tarning*et al.,*2009**[37]	Pharmacokinetic study of AL in pregnancy using capillary plasma samples (n = 103)	Capillary plasma: 391 ng/ml (126–1,600), venous plasma (approx): 310 ng/ml (94–1,364)		AUC_0→∞,_ median (range): 472 μg/ml.h (119–1,261)	
**Piola*et al.,*2010**[25]	Open-label, randomized study of AL *vs.* Q in the 2^nd^ and 3^rd^ trimesters, with pharmacokinetic analysis of venous plasma samples (n = 97)	481 ng/ml (15–3,246)			

*AL* artemether-lumefantrine; *Q* quinine;*90% range; ^†^AUC_60h→∞._

Two of the three efficacy studies of AL during pregnancy reported Day 7 lumefantrine plasma levels, a measure correlated with drug exposure estimates [31]. In particular, Day 7 lumefantrine plasma levels below 280 ng/ml have been related to the risk of therapeutic failure [32]. In the Ugandan study that compared AL and quinine in the second and third trimester of pregnancy and reported an AL cure rate of 98.2% (PCR-corrected at delivery (or Day 42 if later)), venous lumefantrine levels were assessed in 97 women [25]. The median Day 7 lumefantrine concentration of 481 ng/ml was similar to the median concentration of 350 ng/ml previously reported in healthy adults, but the range of values was larger in the pregnant population (15–3,246 *versus* 204–869 ng/ml) [25,31]. Within this population, there were six women with recurrent parasitaemia by delivery (or Day 42 if later) (one recrudescent, two novel, one indeterminate, two non-*falciparum*) and five of these women had Day 7 lumefantrine levels below 280 ng/ml. However, a third of women without recrudescent parasitaemia also had a Day 7 lumefantrine concentration under 280 ng/ml [25].

A pharmacokinetic extension of the Thai study of AL and artesunate during the second and third trimesters, which reported an AL cure rate of 82% (PCR-corrected at delivery (or Day 42 if later), assessed the capillary plasma lumefantrine concentrations in 103 women [37]. This study found no significant differences in Day 7 lumefantrine concentration between women with and without recurrent parasitaemia, although no treatment failures occurred in women with lumefantrine concentrations above 550 ng/ml (approximately 360 ng/ml in venous plasma) [37]. The median capillary plasma lumefantrine exposure (AUC_0→∞_) was 472 μg/ml.h (range 119–1261) and the median Day 7 lumefantrine concentration was 391 ng/ml (range 126–1600), approximately equivalent to a median venous plasma concentration of 310 ng/ml (range 94–1364) and above the 280 ng/ml threshold predictor of treatment failure [37].

## Discussion

Malaria during pregnancy is associated with an increased risk of maternal anaemia, maternal death, abortion, premature labour, low birth weight, and neonatal death [1,7,38]. Control measures recommended in areas of stable transmission to protect against these outcomes include the use of preventive strategies and proper case management, with accurate diagnosis and prompt, effective treatment of malaria and anaemia [6,9].

The current WHO recommendations for the treatment of uncomplicated *falciparum* malaria during pregnancy are: a locally effective ACT (AL, AS-AQ, AS-MQ or AS + SP) in the second and third trimesters of pregnancy, and quinine plus clindamycin or quinine monotherapy in the first trimester [11]. These recommendations were based on available efficacy and safety data for ACT, which were particularly limited in the first trimester.

This review focused on the safety and efficacy of AL during pregnancy, since AL has now been widely available for over ten years in Africa and is used across several continents. It includes over one thousand reports of AL use in pregnancy, with 212 in the first trimester [15-17,20,22-27]. However, it should be noted that the literature review selected English-language publications only, and so there may be relevant reports or studies that are currently unpublished or published in other languages, that have not been included in this article.

The reported studies suggest that AL is generally well tolerated by pregnant women and shows no association between the use of AL in the second and third trimesters of pregnancy and an increased risk of spontaneous abortion, stillbirth or congenital abnormalities when compared with quinine, SP or artesunate [22,25,26]. In comparison with quinine, which is currently recommended throughout pregnancy in some countries, tolerability benefits of AL may improve treatment compliance [25]. Additionally, quinine use in late pregnancy is associated with increased risk of hypoglycaemia [39].

There are fewer available reports on the use of AL by pregnant women in the first trimester, and 156 of these were reported in a recent observational study in Zambia that compared AL and SP [26]. In this study, no differences were detected between first trimester exposures of the two drugs in the occurrence of perinatal death, infant malformation or assessments of infant neurodevelopment. However, seven of 159 foetuses exposed to AL in the first trimester were miscarried, while there were no miscarriages of the 135 foetuses exposed to SP and/or quinine in the first trimester [26]. Although four of the seven events were linked to confounding factors such as infection (Table 2), further investigations of the safety of ACT in the first trimester are recommended.

One factor that may relate to the miscarriages in first trimester AL exposures is the use of presumptive treatment and the risk of treating women without malaria. In this study, malaria cases were predominantly diagnosed based on clinical symptoms without confirmation by microscopy or RDT [26]. Recent studies in animals and humans have suggested that the risk of embryotoxicity from artemisinins may be higher in pregnant women who do not have malaria than those with malaria, as healthy volunteers taking artemisinins have shown greater decreases in reticulocyte count, a possible marker for embryotoxicity, than adults treated for malaria [40]. Another relevant risk factor for miscarriage is malaria infection in the first trimester of pregnancy. Analysis of data collected at the Shoklo Malaria Research Unit between 1986 and 2010 compared the outcomes of 945 confirmed cases of malaria in the first trimester of pregnancy with 16,668 pregnancies without a malaria infection, and showed that malaria infection in the first trimester was associated with increased risk of miscarriage, and this risk was similar in women treated with chloroquine, quinine and artesunate [41].

The available studies suggest that the use of AL in the second and third trimesters of pregnancy is not associated with adverse outcomes. However, to detect any rare adverse events, which may not become apparent in clinical studies, it is important to monitor pregnancy outcomes following all exposures of pregnant women to anti-malarial treatments. This will increase the pool of available safety data for assessment of which treatments are most appropriate for use in pregnancy. There have been calls for anti-malarial pregnancy exposure registries for large-scale data collection [42], and recently the design, feasibility and implementation of an Electronic Perinatal Record system was successfully tested in Lusaka, Zambia [43].

The efficacy of AL has been studied in the second and third trimesters of pregnancy in randomized controlled trials. Studies in Uganda revealed higher efficacy than reports from Thailand [22,23,25]. The efficacy of AL during pregnancy in the two Ugandan trials, but not the Thai trial, is similar to the results from studies in adults and children that have consistently reported AL efficacy of over 95% [44]. The reasons for these differences in cure rate are currently unclear, but several explanations have been suggested. The study in Thailand compared AL and artesunate monotherapy in pregnant women. In this study, fever and parasite clearance were rapid and similar after both treatments. PCR-corrected parasitological cure rates at delivery (or Day 42 if later) were 82.0% for AL and 89.2% for artesunate. Fewer than half of the patients had primary infections at baseline, and for patients with recrudescent infections at baseline, the cure rate with AL was significantly lower than that with artesunate. However, for patients with primary infections or new infections at baseline following a previous episode, cure rates for the two treatments were similar. This is consistent with a previous report of reduced susceptibility of recrudescent infections to quinine, mefloquine and lumefantrine in this area, suggesting that the relatively lower cure rate may be due to relatively resistant parasites under such circumstances [22]. Piola and colleagues proposed that differences in the level of malaria immunity between the sites of the Ugandan and Thai trials could explain the different efficacies of AL in pregnant women, a plausible interpretation given the marked differences in the intensity of malaria transmission. The differences in cure rate could also be explained by the emergence of parasite resistance to artemisinins or lumefantrine at the Thai study site (Thai-Burmese border).

There have also been suggestions that changes in the pharmacokinetics of artemether and lumefantrine during pregnancy may influence the efficacy of AL [21,22,25,29,30,37]. Small changes in AL exposure during pregnancy have been reported [21,25], but these results should be viewed with caution as they involve inter-trial comparisons, small populations, high inter-individual variation in plasma levels and the average plasma levels reported are comparable with data from non-pregnant adults [21,22,25,31-37,45]. Approximately one third of women in the Ugandan and Thai studies had a Day 7 lumefantrine concentration below 280 ng/ml, suggesting that lumefantrine concentration is unlikely to account for the reported differences in AL efficacy between the studies [22,25]. In Tarning *et al.*, no significant differences (p = 0.26) in predicted Day 7 lumefantrine levels were detected between women with and without parasitaemia receiving AL in Thailand. However, it is of note that the predicted median Day 7 lumefantrine concentration in this pregnant population was reported to be lower than previous studies in children and non-pregnant adults in Thailand, Cambodia and the Lao People’s Democratic Republic [37]. These analyses, together with recent results suggesting that DHA clearance may be approximately 42% higher in pregnant women [46,47], support the view that further study of the pharmacokinetics of AL in pregnancy would be of benefit to help maintain confidence in dosing regimens and ensure that pregnant women receive an appropriate treatment regimen. The Malaria in Pregnancy consortium has recently conducted a trial in Uganda comparing the AL pharmacokinetics in pregnant and matched non-pregnant women, and the data from this study are eagerly awaited [48].

Co-morbidities and concomitant medications may also impact on the pharmacokinetics of anti-malarial treatments in children, adults and in pregnant women. There are several reports that HIV infection may influence the outcome of anti-malarial treatments, although the reported effects differ between the studies [49]. One small study in Ethiopia reported reduced clearance of *P. falciparum* by artemisinin in patients with HIV [50]. The potential for drug–drug interactions between both HIV and tuberculosis treatments and anti-malarials has been identified, but only limited pharmacokinetic data are available for some HIV treatments and there are currently no published data available for tuberculosis treatments. The effect of lopinavir/ritonavir (a protease inhibitor combination for treating HIV) on the pharmacokinetics of AL was tested in 13 healthy non-HIV infected adults. This study showed a two- to three-fold increase in lumefantrine exposure (AUC) and small decreases in artemether and DHA exposure (AUC and C_max_) when AL and lopinavir/ritonavir were co-administered [51]. Further studies are required to determine whether these factors may impact on the efficacy and safety of anti-malarials in the general population and during pregnancy.

Guidelines for the case management of malaria vary between countries, including the guidelines for treatment at different stages of pregnancy. Not all countries with endemic malaria have adopted the WHO recommendation to use ACT for uncomplicated *falciparum* malaria during the second and third trimesters of pregnancy. Reasons for recommending alternative treatments such as quinine or SP are likely to differ between countries according to local study data and economic and political considerations. South Africa currently recommends quinine plus clindamycin for the treatment of uncomplicated malaria in pregnancy due to concerns for foetal safety, based on animal studies, and a report of suboptimal lumefantrine absorption during pregnancy [52]. In addition, ACT is not currently recommended during pregnancy in Ethiopia, as local studies were considered necessary to identify those most suitable for use. As part of the President’s Malaria Initiative, there are plans to revise the existing treatment guidelines, and the use of ACT in pregnancy will be considered [53].

The recent publication of new safety data and the results from ongoing studies may impact current national policies. Full publication of a recent study of the safety of DHA-PPQ in the second and third trimester is eagerly awaited. Available data suggest that, in a population of 1,160 pregnant women, there was no increased risk of congenital abnormalities or stillbirths, and so DHA-PPQ may also be a suitable ACT for use in pregnancy [54]. Another trial is currently recruiting subjects in Burkina Faso, Ghana, Malawi and Zambia, with the aim of evaluating the efficacy and safety of four types of ACT (AL, AS-AQ, AS-MQ, and DHA-PPQ) for the treatment of pregnant women with *P. falciparum* malaria (PREGACT study: Safe and Efficacious Artemisinin-based Combination Treatments for African Pregnant Women With Malaria (Clinical Trials identifier NCT00852423)) expected to end in July 2014. There is also a similar trial ongoing in Brazil (Clinical Trials identifier NCT01082731), which is estimated to end in August 2012, and a pharmacovigilance study in Africa (Clinical Trials identifier NCT01232530) expected to end in October 2013.

## Conclusions

The use of AL for the treatment of uncomplicated *P. falciparum* malaria in pregnancy is supported by a large body of evidence. In the second and third trimesters, the available safety data support the use of AL, but further studies to assess the safety of AL in the first trimester are recommended. Since quinine with clindamycin is the currently recommended treatment during the first trimester, studies comparing the safety of AL *versus* quinine and clindamycin may be most appropriate. AL may be preferable to quinine in the second and third trimesters as AL efficacy is non-inferior to quinine, but is associated with fewer adverse events. There are some reports that suggest there may be a change in AL pharmacokinetics in pregnancy, although the impact on efficacy and safety needs to be studied further, especially since the majority of studies in pregnancy report high cure rates and adequate tolerability. Direct comparisons of drug levels and efficacy between pregnant and non-pregnant women are needed to separate any pharmacokinetic change in pregnancy from other factors such as the type of infection (novel or recrudescent), background immunity and parasite sensitivity.

The WHO recommendation for the use of appropriate ACT in the second and third trimesters of pregnancy is supported by the currently available data and the universal implementation of these recommendations throughout the African WHO region should occur. There is also need for further safety studies on the outcomes of ACT use during pregnancy and in particular, comparisons between the efficacy and safety of ACT and quinine with clindamycin during the first trimester.

## Abbreviations

ACT: Artemisinin-based combination therapy; AL: Artemether-lumefantrine; AS-AQ: Artesunate-amodiaquine; AS-MQ: Artesunate-mefloquine; AS + SP: Artesunate + sulphadoxine-pyrimethamine; AUC: Area under the curve; DHA: Dihydroartemisinin; DHA-PPQ: Dihydroartemisinin-piperaquine; IPTp: Intermittent preventive treatment in pregnancy; RDT: Rapid diagnostic test; SP: Sulphadoxine-pyrimethamine; WHO: World Health Organization.

## Competing interests

CM, KK, HUO and EJ have no competing interests. UDA has received research funding from Sigma Tau and Sanofi-Aventis. He has also received travel grants from Sigma Tau and Novartis. KH is an employee of Novartis Pharmaceuticals Corporation, East Hanover, NJ, USA.

## Authors’ contributions

All authors met International Committee of Medical Journal Editors criteria for authorship. All authors contributed to the development of the outline, revised the manuscript critically, and read and approved the final manuscript.

## Supplementary Material

Additional file 1 **Ovid search strategy.** Ovid was used to search the EMBASE, MEDLINE, BIOSIS (included In-Process and Other Non-indexed Citations, Daily Update) and Cochrane (included Cochrane Database of Systematic Reviews, APC Journal Club, Database of Abstracts of Reviews of Effects, Cochrane Central Register of Controlled Trials, Cochrane Methodology Register, Health Technology Assessment, NHS Economic Evaluation Database) databases between 1948 and 2011. Six queries were performed using the search terms shown. *Search for the term shown with or without any number of additional letters; ?Search for the term shown with or without one additional letter. Click here for file

Additional file 2 **Results of literature search: Number of references identified for each query and database.** EMBASE, MEDLINE, BIOSIS and Cochrane were searched on 16 December, 2011. Malaria in Pregnancy consortium library, Clinicaltrials.gov and WHO ICTRP were searched on 8 December, 2011 and TrialTrove was searched on 14 December, 2011. ICTRP, International Clinical Trials Registry Platform. Click here for file

Additional file 3 400 unique references recovered in the literature search. Click here for file

Additional file 4 **Publications reporting on the safety, efficacy and pharmacokinetics of artemether and artemether-lumefantrine in pregnancy, including the number of exposures during pregnancy.** Sixteen articles were identified by the literature search, all reporting on pregnancies exposed to artemether or artemether-lumefantrine. The number of pregnancies exposed to artemether or artemether-lumefantrine are reported, and it is noted where exposures have been previously reported. AL, artemether-lumefantrine; AE, adverse events; SAE, serious adverse events; SP, sulphadoxine-pyrimethamine; ACT, artemisinin-based combination therapies; ^†^National treatment policy is quinine throughout pregnancy [55-57]; ^†^National treatment policy is quinine in the first trimester and ACT in the second and third trimesters of pregnancy [55,58,59]; ^§^National treatment policy is quinine in the first trimester and SP in the second and third trimesters of pregnancy; Benin,^†^ Burkina Faso,^†^ Cameroon,^†^ DRC,^†^ Gabon,^†^ Mozambique,^†^ Mali,^†^ Thailand,^†^ Ghana,^‡^ Kenya,^‡^ Malawi,^‡^ Nigeria,^‡^ Sudan,^‡^ Tanzania,^‡^ Uganda,^‡^ Zambia,^§^[26,55,57-59]; ^#^McGready *et al.*, 2001 [17] includes all of the artemether exposures reported across three publications. To avoid duplication of pregnancy exposures the other two reports have not been included [18,19]; ^¶^Included women with a pregnancy in the last 12 months that lasted until at least the third trimester. Current pregnancies were excluded; ^††^Mali,^†^ Mozambique,^†^ Thailand,^†^ Kenya,^‡^ Nigeria,^‡^ Sudan,^‡^ Tanzania,^‡^ Zambia^§^[26,55,57,59]; ^‡‡^National treatment policy is quinine/AL [60]. Wang, 1989 [20], Sowunmi *et al.,* 1998 [15], McGready *et al.*, 2001 [17], Adam *et al.,* 2004 [16], McGready *et al.,* 2006 [21], Dellicour *et al.,* 2007 [14], McGready *et al.*, 2008 [22], Kaye *et al.*, 2008 [23], Orton *et al.*, 2008 [61], Adam *et al.,* 2009 [24], Tarning *et al.,* 2009 [37], Piola *et al.,* 2010 [25], Manyando *et al.,* 2010 [26], McGready *et al.,* 2011 [29], Sangaré *et al.,* 2011 [27], Wilby and Ensom, 2011 [30]. Click here for file
