# Supplementary material for: A systematic review of the safety and efficacy of artemether-lumefantrine against uncomplicated Plasmodium falciparum malaria during pregnancy
Source: Malar J. 2012 May 1;11:141. doi: 10.1186/1475-2875-11-141 (PMC3405476; doi:10.1186/1475-2875-11-141)
Supplement: Additional file 1 — Ovid search strategy. Ovid was used to search the EMBASE, MEDLINE, BIOSIS (included In-Process and Other Non-indexed Citations, Daily Update) and Cochrane (included Cochrane Database of Systematic Reviews, APC Journal Club, Database of Abstracts of Reviews of Effects, Cochrane Central Register of Controlled Trials, Cochrane Methodology Register, Health Technology Assessment, NHS Economic Evaluation Database) databases between 1948 and 2011. Six queries were performed using the search terms shown. *Search for the term shown with or without any number of additional letters; ?Search for the term shown with or without one additional letter. [file 1475-2875-11-141-S1.doc]

| **Query** | **Search name** | **Search terms** |
| --- | --- | --- |
| **1** | Artemether-lumefantrine AND pregnancy | Artemether lumefantrin?, artemether benflumetol, cgp 56697, cgp56697, co artem, coartem, coa 566, coa566, coartemether, riamet  pregnanc*, pregnant |
| **2** | Artemether AND pregnancy | Artemether, aids 008344, aids008344, artemetero, artemetherum, artemisininelactol methyl ether, artemisininelactolmethylether, artenam, arthemether, cgp 56696, cgp56696, dihydroartemisinin? methyl ether, methyl dihydroartemisinin sm 224, paluther  pregnanc*, pregnant |
| **3** | Lumefantrine AND pregnancy | Lumefantrin?, benflumetol, cgp 56695, cgp56695  pregnanc*, pregnant |
| **4** | Artemisinins AND pregnancy | Artemisinin?, arteannuin, artemisin?, ching hao su, chinghaosu, ginghaosu, qinghaosu, quinghaosu, quinhaosu |
| **5** | Artemisinin-based Combination Therapy (ACT) AND pregnancy AND malaria | Artemisinin? combination, artemisinin? combine?  pregnanc*, pregnant  malaria |
| **6** | Artemisinin-based Combination Therapy (ACT) AND pregnancy AND *Plasmodium falciparum* | Artemisinin? combination, artemisinin? combine?  pregnanc*, pregnant  plasmod?, falcip* |
