# Supplementary material for: A systematic review of the safety and efficacy of artemether-lumefantrine against uncomplicated Plasmodium falciparum malaria during pregnancy
Source: Malar J. 2012 May 1;11:141. doi: 10.1186/1475-2875-11-141 (PMC3405476; doi:10.1186/1475-2875-11-141)
Supplement: Additional file 2 — Results of literature search: Number of references identified for each query and database. EMBASE, MEDLINE, BIOSIS and Cochrane were searched on 16 December, 2011. Malaria in Pregnancy consortium library, Clinicaltrials.gov and WHO ICTRP were searched on 8 December, 2011 and TrialTrove was searched on 14 December, 2011. ICTRP, International Clinical Trials Registry Platform. [file 1475-2875-11-141-S2.doc]

|  | Query | **References identified through database searching** | | | | | **Clinical trial records identified through database searching** | | | |
| --- | --- | --- | --- | --- | --- | --- | --- | --- | --- | --- |
|  |  | EMBASE | Medline | BIOSIS | Cochrane Databases | Malaria in Pregnancy | Clinicaltrials.gov | WHO ICTRP | Malaria in Pregnancy | TrialTrove |
| 1 | Artemether-lumefantrine AND pregnancy | 113 | 40 | 18 | 20 | 16 | 6 | 7 | 6 | 3 |
| 2 | Artemether AND pregnancy | 104 | 25 | 15 | 3 | 37 | 6 | 7 | 6 | 3 |
| 3 | Lumefantrine AND pregnancy | 13 | 2 | 1 | 0 | 20 | 7 | 7 | 7 | 3 |
| 4 | Artemisinins AND pregnancy | 166 | 145 | 56 | 26 | 79 | 12 | 4 | 7 | 2 |
| 5 | Artemisinin-based Combination Therapy (ACT) AND pregnancy AND malaria | 0 | 0 | 0 | 0 | 38 | 6 | 0 | 5 | - |
| 6 | Artemisinin-based Combination Therapy (ACT) AND pregnancy AND *Plasmodium falciparum* | 0 | 0 | 0 | 0 | 11 | 2 | 0 | 1 | - |
| 7 | Artemether or artemether + lumefantrine or artemether + lumefantrine, Cipla AND pregnancy | - | - | - | - | - | - | - | - | 5 |
| 8 | Artemether or artemether + lumefantrine or artemether + lumefantrine, Cipla AND pregnancy AND malaria | - | - | - | - | - | - | - | - | 5 |
| 9 | Artemether or artemether + lumefantrine or artemether + lumefantrine, Cipla AND pregnancy AND *Plasmodium falciparum* | - | - | - | - | - | - | - | - | 1 |
