# Supplementary material for: A systematic review of the safety and efficacy of artemether-lumefantrine against uncomplicated Plasmodium falciparum malaria during pregnancy
Source: Malar J. 2012 May 1;11:141. doi: 10.1186/1475-2875-11-141 (PMC3405476; doi:10.1186/1475-2875-11-141)
Supplement: Additional file 3 — 400 unique references recovered in the literature search. [file 1475-2875-11-141-S3.doc]

| Abba K, Deeks JJ, Olliaro PL, et al (2010) Rapid diagnostic tests for diagnosing uncomplicated malaria in endemic countries Systematic Review. Cochrane Database of Systematic Reviews; 8: |
| --- |
| Abdelrahim II, Adam I, Elghazali G, Gustafsson LL, Elbashir MI, Mirghani RA. Pharmacokinetics of quinine and its metabolites in pregnant Sudanese women with uncomplicated Plasmodium falciparum malaria. J Clin Pharm Ther. 2007 Feb;32(1):15-9. PubMed PMID: 17286785. |
| Abramowicz M, Zuccotti G, Pflomm J-M, et al (2009) Artemether-lumefantrine (Coartem) for treatment of malaria. Medical Letter on Drugs and Therapeutics; 51 (1321):75-76. |
| Achan J, Talisuna AO, Erhart A, Yeka A, Tibenderana JK, Baliraine FN, Rosenthal PJ, D'Alessandro U. Quinine, an old anti-malarial drug in a modern world: role in the treatment of malaria. Malar J. 2011 May 24;10:144. Review. PubMed PMID: 21609473; PubMed Central PMCID: PMC3121651. |
| Adam I, Ali DM, Abdalla MA. Artesunate plus sulfadoxine-pyrimethamine in the treatment of uncomplicated Plasmodium falciparum malaria during pregnancy in eastern Sudan. Trans Royal Soc Trop Med Hyg 2006;100(7);632-635 |
| Adam I, Elhassan EM, Omer EM, Abdulla MA, Mahgoub HM, Adam GK. Safety of artemisinins during early pregnancy, assessed in 62 Sudanese women. Ann Trop Med Parasitol. 2009 Apr;103(3):205-10. PubMed PMID: 19341535. |
| Adam I, Elwasila E, Mohammed Ali DA, Elansari E, Elbashir MI. Artemether in the treatment of falciparum malaria during pregnancy in eastern Sudan. Trans R Soc Trop Med Hyg. 2004 Sep;98(9):509-13. PubMed PMID: 15251398. |
| Adam I, Karsany MS (2008) Case report: Rift valley fever with vertical transmission in a pregnant Sudanese woman. Journal of Medical Virology; 80 (5):929. |
| Adam I, Mirghani OA, Saed OK, et al (2004) Quinine therapy in severe Plasmodium falciparum malaria during pregnancy in Sudan. Eastern Mediterranean Health Journal; 10 (1-2):159-166. |
| Ades V. Safety, pharmacokinetics and efficacy of artemisinins in pregnancy. Infectious Disease Reports 2011;3:e8 |
| Agan TU, Ekabua JE, Iklaki CU, et al (2010) Prevalence of asymptomatic malaria parasitaemia. Asian Pacific Journal of Tropical Medicine; 3 (1):51-54. |
| Agrawal VK (2008) Plasmodium falciparum containment strategy. Medical Journal Armed Forces India; 64 (1):57-60. |
| Ahmad R, Zareen (1998) Malaria in pregnancy. Journal of the College of Physicians and Surgeons Pakistan; 8 (2):82-85. |
| Ahorlu CK, Koram KA, Seakey AK, et al (2009) Effectiveness of combined intermittent preventive treatment for children and timely home treatment for malaria control. Malaria journal; 8:292. |
| Alecrim WD, Espinosa FEM, Alecrim MGC (2000) Plasmodium falciparum infection in the pregnant patient. Infectious Disease Clinics of North America; 14 (1):83-95. |
| Ali A, Elghazali G, Elagib I, et al (2006) Pharmacokinetics of quinine and its metabolites in pregnant women infected with Plasmodium falciparum. Drug Metabolism Reviews; 38 (Suppl. 1):70-71. |
| Alifrangis M, Lusingu JP, Mmbando B, et al (2009) Five-year surveillance of molecular markers of Plasmodium falciparum antimalarial drug resistance in Korogwe District, Tanzania: accumulation of the 581G mutation in the P. falciparum dihydropteroate synthase gene. The American journal of tropical medicine and hygiene; 80 (4):523-527. |
| AlKadi HO. Antimalarial drug toxicity: a review. Chemotherapy. 2007;53(6):385-91. Epub 2007 Oct 12. Review. PubMed PMID: 17934257. |
| Alvarez A, Al-Khan A, Apuzzio JJ. Malaria in pregnancy. Infectious diseases in obstetrics and gynecology 2005;13(4):229-236 |
| Amzat J. Assessing the progress of malaria control in Nigeria. World Health Popul. 2011;12(3):42-51. PubMed PMID: 21677528. |
| Andriantsoanirina V, Bouchier C, Tichit M, et al (2010) Origins of the recent emergence of Plasmodium falciparum pyrimethamine resistance alleles in Madagascar. Antimicrobial Agents and Chemotherapy; 54 (6):2323-2329. |
| Anon (2000) Severe falciparum malaria. Transactions of the Royal Society of Tropical Medicine and Hygiene; 94 (SUPPL. 1):S1-S90. |
| Anon (2004) The risks and benefits of treatments must be considered when treating or preventing malaria. Drugs and Therapy Perspectives; 20 (12):22-25. |
| Anon (2005) Unique partnership supports a malaria capacity building program in Zambia. International Nursing Review; 52 (2):84-90. |
| Artemisinin derivatives and malaria: useful, in combination with other antimalarials. Prescrire Int. 2008 Aug;17(96):162-8. PubMed PMID: 19492494. |
| Ashley E, McGready R, Proux S, et al (2006) Malaria. Review 78 refs. Travel Medicine & Infectious Disease; 4 (3-4):159-173. |
| Ashley E, McGready R, Proux S, Nosten F. Malaria. Travel Med Infect Dis. 2006 May-Jul;4(3-4):159-73. Epub 2005 Sep 19. Review. PubMed PMID: 16887738. |
| Ashley EA, White NJ. Artemisinin-based combinations. Curr Opin Infect Dis. 2005 Dec;18(6):531-6. Review. PubMed PMID: 16258328. |
| Athip N, Suthinee B, Sueptrakool W (2004) Effect of antimalarial drugs on cytotrophoblast survival. American Journal of Reproductive Immunology; 52 (Suppl. 1):44. |
| Attioua B, Weniger B, Chabert P (2007) Antiplasmodial activity of constituents isolated from Croton lobatus. Pharmaceutical Biology; 45 (4):263-266. |
| Ayoola,H.N., Daniel,E.A., Oladeji,I. Pattern of malaria presentation and treatment at Obisesan Naval Medical Centre Lagos-Nigeria. European Journal of Scientific Research 2009; 1:120-127 |
| Baker L (2010) A practical guide to the management of malaria in South Africa - 2010: Adapted from Manguzi Hospital Guidelines. SA Pharmaceutical Journal; 77 (3):28-32. |
| Balasegaram M, Dejene S, Tinnemann P, Perkins S, Davidson R. Examples of tropical disease control in the humanitarian medical programmes of MSF and Merlin. Trans R Soc Trop Med Hyg. 2006 Apr;100(4):327-34. Epub 2005 Nov 11. PubMed PMID: 16289631. |
| Baniecki ML, Wirth DF, Clardy J (2007) High-throughput Plasmodium falciparum growth assay for malaria drug discovery. Antimicrobial Agents and Chemotherapy; 51 (2):716-723. |
| Barnes KI, Watkins WM, White NJ (2008) Antimalarial dosing regimens and drug resistance. Trends in Parasitology; 24 (3):127-134. |
| Belay M, Deressa W (2008) Use of insecticide treated nets by pregnant women and associated factors in a pre-dominantly rural population in northern Ethiopia. Tropical Medicine and International Health; 13 (10):1303-1313. |
| Bell D, Winstanley P (2004) Current issues in the treatment of uncomplicated malaria in Africa. British Medical Bulletin; 71:29-43. |
| Bello SO, Chika A, Bello AY (2010) Is chloroquine better than artemisinin combination therapy as first line treatment in adult nigerians with uncomplicated malaria? - A cost effectiveness analysis. African Journal of Infectious Diseases; 4 (2):29-42. |
| Benowitz NL, Hukkanen J, Jacob IP (2009) Nicotine chemistry, metabolism, kinetics and biomarkers. Nicotine Psychopharmacology; Handbook of Experimental Pharmacology. 192:29-60. |
| Bhattarai A, Ali AS, Kachur SP, et al (2007) Impact of artemisinin-based combination therapy and insecticide-treated nets on malaria burden in Zanzibar. PLoS Medicine; 4 (11):1784-1790. |
| Bhattarai A, Maini-Thapar M, Ali AS, Björkman A. Amodiaquine during pregnancy. Lancet Infect Dis. 2004 Dec;4(12):721-2; discussion 722. PubMed PMID: 15567117. |
| Bhatti MA, Azharuddin M, Bhatti S, Islam M, Khan MA. Malaria and pregnancy: the perspective in Pakistan. J Pak Med Assoc. 2007 Jan;57(1):15-8. PubMed PMID: 17319413. |
| Boareto AC, Muller JC, Bufalo AC, Botelho GG, de Araujo SL, Foglio MA, de Morais RN, Dalsenter PR. Toxicity of artemisinin [Artemisia annua L.] in two different periods of pregnancy in Wistar rats. Reprod Toxicol. 2008 Feb;25(2):239-46. Epub 2007 Nov 17. PubMed PMID: 18191938. |
| Bojang KA, Akor F, Conteh L, et al (2011) Two strategies for the delivery of IPTc in an area of seasonal malaria transmission in the Gambia: A randomised controlled trial. PLoS Medicine; 8 (2): |
| Bounyasong S (2001) Randomized trial of artesunate and mefloquine in comparison with quinine sulfate to treat P. falciparum malaria pregnant women. Journal of the Medical Association of Thailand = Chotmaihet thangphaet; 84 (9):1289-1299. |
| Brabin BJ, Warsame M, Uddenfeldt-Wort U, Dellicour S, Hill J, Gies S. Monitoring and evaluation of malaria in pregnancy - developing a rational basis for control. Malar J. 2008 Dec 11;7 Suppl 1:S6. Review. Erratum in: Malar J. 2009;8:146. Wasame, Marian [corrected to Warsame, Marian]. PubMed PMID: 19091040; PubMed Central PMCID: PMC2604870. |
| Breman JG, Alilio MS, Mills A. Conquering the intolerable burden of malaria: what's new, what's needed: a summary. Am J Trop Med Hyg. 2004 Aug;71(2 Suppl):1-15. Review. PubMed PMID: 15331814. |
| Breman JG. Eradicating malaria. Sci Prog. 2009;92(Pt 1):1-38. Review. PubMed PMID: 19544698. |
| Brentlinger PE, Behrens CB, Micek MA. Challenges in the concurrent management of malaria and HIV in pregnancy in sub-Saharan Africa. The Lancet Infectious Diseases 2006;6(2):100-111 |
| Briand V, Badaut C, Cot M (2009) Placental malaria, maternal HIV infection and infant morbidity. Annals of Tropical Paediatrics; 29 (2):71-83. |
| Briand V, Cottrell G, Massougbodji A, et al (2007) Intermittent preventive treatment for the prevention of malaria during pregnancy in high transmission areas. Malaria Journal; 6: |
| Brooks MI, Singh N, Hamer DH (2008) Control measures for malaria in pregnancy in India. Indian Journal of Medical Research; 128 (3):246-253. |
| Buffet PA, Briand V, Renia L, et al (2008) Intermittent preventive antimalarial treatment to children (IPTc): firebreak or fire trap? Trends in Parasitology; 24 (11):482-485. |
| Bukirwa H, Garner P, Critchley JA (2011) Chlorproguanil-dapsone for treating uncomplicated malaria Systematic Review. Cochrane Database of Systematic Reviews; 1: |
| Burrows JN, Chibale K, Wells TNC (2011) The state of the art in anti-malarial drug discovery and development. Current Topics in Medicinal Chemistry; 11 (10):1226-1254. |
| Byakika-Kibwika P, Lamorde M, Mayanja-Kizza H, et al (2010) Update on the efficacy, effectiveness and safety of artemether-lumefantrine combination therapy for treatment of uncomplicated malaria. Therapeutics and Clinical Risk Management; 6 (1):11-20. |
| Canter A, Twombly J (2010) Case study: Novartis malaria initiative - How innovative alliances produced a winning sourcing partnership. Contract Pharma; (7): |
| Caplan A (2004) Malaria initiative 2. Lancet; 364 (9447):1752. |
| Carrara VI, Sirilak S, Thonglairuam J, et al (2006) Deployment of early diagnosis and mefloquine-artesunate treatment of falciparum malaria in Thailand: the Tak Malaria Initiative. PLos Medicine; 3 (6):e183. |
| Carroll ID. Avoiding travel-related infections during pregnancy. Contemporary OB/GYN 2005:34-43 |
| Carvalho BO, Matsuda JS, Luz SL, et al (2011) Gestational malaria associated to Plasmodium vivax and Plasmodium falciparum placental mixed-infection followed by foetal loss: A case report from an unstable transmission area in Brazil. Malaria Journal; 10: |
| Castelli F, Odolini S, Autino B, et al (2010) Malaria prophylaxis: A comprehensive review. Pharmaceuticals; 3 (10):3212-3239. |
| Ceesay SJ, Casals-Pascual C, Erskine J, et al (2008) Changes in malaria indices between 1999 and 2007 in The Gambia: a retrospective analysis. The Lancet; 372 (9649):1545-1554. |
| Centers for Disease Control and Prevention (CDC). Grand rounds: The opportunity for and challenges to malaria eradication. MMWR Morb Mortal Wkly Rep. 2011 Apr 22;60(15):476-80. PubMed PMID: 21508924. |
| Chanda P, Hamainza B, Mulenga S, et al (2009) Early results of integrated malaria control and implications for the management of fever in under-five children at a peripheral health facility: A case study of Chongwe rural health centre in Zambia. Malaria journal; 8 (1): |
| Chanda P, Masiye F, Chitah BM, et al (2007) A cost-effectiveness analysis of artemether lumefantrine for treatment of uncomplicated malaria in Zambia. Malaria journal; 6: |
| Chattopadhyay R, Mahajan B, Kumar S (2007) Assessment of safety of the major antimalarial drugs. Expert Opinion on Drug Safety; 6 (5):505-521. |
| Checchi F, Roddy P, Kamara S, Williams A, Morineau G, Wurie AR, Hora B, Lamotte N, Baerwaldt T, Heinzelmann A, Danks A, Pinoges L, Oloo A, Durand R, Ranford-Cartwright L, Smet M; Sierra Leone Antimalarial Efficacy Study Collaboration. Evidence basis for antimalarial policy change in Sierra Leone: five in vivo efficacy studies of chloroquine, sulphadoxine-pyrimethamine and amodiaquine. Trop Med Int Health. 2005 Feb;10(2):146-53. PubMed PMID: 15679557. |
| Chen X-G, Wu K, Lun Z-R (2005) Toxoplasmosis researches in China. Chinese Medical Journal; 118 (12):1015-1021. |
| Chico RM, Chandramohan, D. Azithromycin plus chloroquine: combination therapy for protection against malaria and sexually transmitted infections in pregnancy. Expert Opinion on Drug Metabolism & Toxicology 2011;7(9):1153-1167 |
| Chico RM, Chandramohan. Quinine for the treatment of malaria in pregnancy. The Lancet Infectious Diseases 2010;10(3):140-141 |
| Chijioke CP. Clinical pharmacology of malaria. Orient Journal of Medicine 2004; 16(2):59-69 |
| Clark IA, Chaudhri G. Tumor necrosis factor in malaria-induced abortion. Am J Trop Med Hyg. 1988 Sep;39(3):246-9. PubMed PMID: 3177738. |
| Clark RL, Arima A, Makori N, et al (2008) Artesunate: developmental toxicity and toxicokinetics in monkeys. Birth Defects Research; 83 (4):418-434. |
| Clark RL, Brannen KC, Sanders JE, Hoberman AM. Artesunate and artelinic acid: association of embryotoxicity, reticulocytopenia, and delayed stimulation of hematopoiesis in pregnant rats. Birth Defects Res B Dev Reprod Toxicol. 2011 Feb;92(1):52-68. doi: 10.1002/bdrb.20282. PubMed PMID: 21312322. |
| Clark RL, Lerman SA, Cox EM, Gristwood WE, White TE. Developmental toxicity of artesunate in the rat: comparison to other artemisinins, comparison of embryotoxicity and kinetics by oral and intravenous routes, and relationship to maternal reticulocyte count. Birth Defects Res B Dev Reprod Toxicol. 2008 Aug;83(4):397-406. PubMed PMID: 18702118. |
| Clark RL, White TE, A Clode S, Gaunt I, Winstanley P, Ward SA. Developmental toxicity of artesunate and an artesunate combination in the rat and rabbit. Birth Defects Res B Dev Reprod Toxicol. 2004 Dec;71(6):380-94. PubMed PMID: 15617018. |
| Clark RL. Embryotoxicity of the artemisinin antimalarials and potential consequences for use in women in the first trimester. Reprod Toxicol. 2009 Nov;28(3):285-96. Epub 2009 May 15. Review. PubMed PMID: 19447170. |
| Coldren RL, Jongsakul K, Vayakornvichit S, et al (2007) Apparent relapse of imported Plasmodium ovale malaria in a pregnant woman. American Journal of Tropical Medicine and Hygiene; 77 (5):992-994. |
| Coll O, Menendez C Botet F, Dayal R, WAPM Perinatal Infections Working Group. Treatment and prevention of malaria in pregnancy and newborn. J Perinat Med 2006;36:15-29 |
| Collignon P, Hehir J, Mitchell D. Successful treatment of falciparum malaria in pregnancy with mefloquine. Lancet 1989; 8644:967 PubMed ID 2565466 |
| Craft JC (2008) Challenges facing drug development for malaria. Current Opinion in Microbiology; 11 (5):428-433. |
| Crawley J, Hill J, Yartey J, et al (2007) From evidence to action? Challenges to policy change and programme delivery for malaria in pregnancy. Lancet Infectious Diseases; 7 (2):145-155. |
| Crawley J, Nahlen B (2004) Prevention and treatment of malaria in young African children. Seminars in Pediatric Infectious Diseases; 15 (3):169-180. |
| Crockett M, Kain KC (2007) Tafenoquine: A promising new antimalarial agent. Expert Opinion on Investigational Drugs; 16 (5):705-715. |
| Cui L, Su XZ. Discovery, mechanisms of action and combination therapy of artemisinin. Expert Rev Anti Infect Ther. 2009 Oct;7(8):999-1013. Review. PubMed PMID: 19803708; PubMed Central PMCID: PMC2778258. |
| Dahlstrom S, Veiga MI, Martensson A, et al (2009) Polymorphism in Pfmrp1 (Plasmodium falciparum multidrug resistance protein 1) amino acid 1466 associated with resistance to sulfadoxine-pyrimethamine treatment. Antimicrobial Agents and Chemotherapy; 53 (6):2553-2556. |
| D'Alessandro S, Gelati M, Basilico N, Parati EA, Haynes RK, Taramelli D. Differential effects on angiogenesis of two antimalarial compounds, dihydroartemisinin and artemisone: implications for embryotoxicity. Toxicology. 2007 Nov 20;241(1-2):66-74. Epub 2007 Aug 19. PubMed PMID: 17897768. |
| d'Alessandro U (1999) A rational approach to malaria control in pregnancy in sub-Saharan Africa: The need for a link between scientific research and public-health interventions. Annals of Tropical Medicine and Parasitology; 93 (SUPPL. 1):S75-S77. |
| d'Alessandro U, ter Kuile FO (2006) Amodiaquine, malaria, pregnancy: the old new drug. Lancet; 368 (9544):1306-1307. |
| D'Alessandro U. Existing antimalarial agents and malaria-treatment strategies. Expert Opin Pharmacother. 2009 Jun;10(8):1291-306. Review. PubMed PMID: 19463069. |
| D'alessandro U. Progress in the development of piperaquine combinations for the treatment of malaria. Curr Opin Infect Dis. 2009 Dec;22(6):588-92. Review. PubMed PMID: 19773652. |
| Davey C (1997) Wormseed warning 4. Pharmaceutical Journal; 258 (6947):840. |
| Davis TME, Mueller I, Rogerson SJ. Prevention and treatment of malaria in pregnancy. Future Microbiology 2010;5(10):1599-1613 |
| Day N, Dondorp AM. The management of patients with severe malaria. Am J Trop Med Hyg. 2007 Dec;77(6 Suppl):29-35. Review. PubMed PMID: 18165472. |
| De Las Heras FG (2011) Overview of neglected tropical diseases. Third World Diseases; Topics in Medicinal Chemistry. 7:1-46. |
| De MS, von SL, Deen JL, et al (2001) Community perceptions of a mass administration of an antimalarial drug combination in The Gambia. Tropical Medicine & International Health; 6 (6):442-448. |
| Deen JL, von Seidlein L, Pinder M, Walraven GEL, Greenwood BM. The safety of the combination artesunate and pyrimethamine-sulfadoxine given during pregnancy. Trans Royal Soc Trop Med Hyg 2001;95(4):424-428 |
| Dellicour S, Hall S, Chandramohan D, Greenwood B. The safety of artemisinins during pregnancy: a pressing question. Malar J. 2007 Feb 14;6:15. Review. PubMed PMID: 17300719; PubMed Central PMCID: PMC1802871. |
| Dellicour S, ter Kuile FO, Stergachis A. Pregnancy exposure registries for assessing antimalarial drug safety in pregnancy in malaria endemic countries. PLoS Medicine 2008;9:e187. PubMed ID 18788893 |
| Dharia NV, Plouffe D, Bopp SER, et al (2010) Genome scanning of Amazonian Plasmodium falciparum shows subtelomeric instability and clindamycin-resistant parasites. Genome Research; 20 (11):1534-1544. |
| Djimde A, Lefevre G (2009) Understanding the pharmacokinetics of Coartem. Malaria journal; 8 Suppl 1:S4. |
| Doherty T, Pinder M (1997) Malaria prevention: Where do we go from here? Current Opinion in Infectious Diseases; 10 (6):466-468. |
| Dondorp AM, Day NPJ (2007) The treatment of severe malaria. Transactions of the Royal Society of Tropical Medicine and Hygiene; 101 (7):633-634. |
| Dorman E, Shulman C (2000) Malaria in pregnancy. Current Obstetrics and Gynaecology; 10 (4):183-189. |
| Druml C, Kremsner PG (2007) The fight against malaria - This month in Vienna. Wiener Klinische Wochenschrift; 119 (9-10):269-270. |
| Duffy PE, Fried M (2005) Malaria in the pregnant woman. Current Topics in Microbiology and Immunology; 295:169-200. |
| Duke T (2011) Randomised trials in child health in developing countries 2011. Annals of Tropical Paediatrics; 31 (4):283-285. |
| Ejiofor JI, Kwanashie HO, Anuka JA. Some pregnancy-related effects of artemether in laboratory animals. Pharmacology. 2006;77(4):166-70. Epub 2006 Jul 12. PubMed PMID: 16837780. |
| El-Dakdoky MH. Evaluation of the developmental toxicity of artemether during different phases of rat pregnancy. Food Chem Toxicol. 2009 Jul;47(7):1437-41. Epub 2009 Mar 28. PubMed PMID: 19332101. |
| Enato EFO, Okhamafe AO (2005) Plasmodium falciparum malaria and antimalarial interventions in sub-Saharan Africa: Challenges and opportunities. African Journal of Biotechnology; 4 (13 SPEC. ISS.):1598-1605. |
| Falade C, Manyando C. Safety profile of Coartem: the evidence base. Malar J. 2009 Oct 12;8 Suppl 1:S6. Review. PubMed PMID: 19818173; PubMed Central PMCID: PMC2760241. |
| Fehintola FA, Akinyinka OO, Adewole IF, et al (2011) Drug interactions in the treatment and chemoprophylaxis of malaria in HIV infected individuals in sub Saharan Africa. Current Drug Metabolism; 12 (1):51-56. |
| Fenwick A, Webster JP (2006) Schistosomiasis: Challenges for control, treatment and drug resistance. Current Opinion in Infectious Diseases; 19 (6):577-582. |
| Fernandes N, Figueiredo P, do Rosario VE, et al (2007) Analysis of sulphadoxine/pyrimethamine resistance-conferring mutations of Plasmodium falciparum from Mozambique reveals the absence of the dihydrofolate reductase 164L mutant. Malaria Journal; 6:35. |
| Figueiredo P, Benchimol C, Lopes D, et al (2008) Prevalence of pfmdr1, pfcrt, pfdhfr and pfdhps mutations associated with drug resistance, in Luanda, Angola. Malaria journal; 7:236. |
| Finaurini S, Ronzoni L, Colancecco A, et al (2010) Selective toxicity of dihydroartemisinin on human CD34+erythroid cell differentiation. Toxicology; 276 (2):128-134. |
| Flateau C, Le LG, Pialoux G (2011) Consequences of HIV infection on malaria and therapeutic implications: A systematic review. The Lancet Infectious Diseases; 11 (7):541-556. |
| Flick K, Scholander C, Chen Q, Fernandez V, Pouvelle B, Gysin J, Wahlgren M. Role of nonimmune IgG bound to PfEMP1 in placental malaria. Science. 2001 Sep 14;293(5537):2098-100. PubMed PMID: 11557894. |
| Franco-Paredes C, Santos-Preciado JI (2006) Problem pathogens: Prevention of malaria in travellers. Lancet Infectious Diseases; 6 (3):139-149. |
| Freedman DO (2008) The author replies. New England Journal of Medicine; 359 (21):2293-2294. |
| Friis H, Gomo E, Koestel P, Ndhlovu P, Nyazema N, Krarup H, Michaelsen KF. HIV and other predictors of serum beta-carotene and retinol in pregnancy: a cross-sectional study in Zimbabwe. Am J Clin Nutr. 2001 Jun;73(6):1058-65. PubMed PMID: 11382660. |
| Friis H, Gomo E, Koestel P, Ndhlovu P, Nyazema N, Krarup H, Michaelsen KF. HIV and other predictors of serum folate, serum ferritin, and hemoglobin in pregnancy: a cross-sectional study in Zimbabwe. Am J Clin Nutr. 2001 Jun;73(6):1066-73. PubMed PMID: 11382661. |
| Friis H, Gomo E, Nyazema N, Ndhlovu P, Kaestel P, Krarup H, Michaelsen KF. HIV-1 viral load and elevated serum alpha(1)-antichymotrypsin are independent predictors of body composition in pregnant Zimbabwean women. J Nutr. 2002 Dec;132(12):3747-53. PubMed PMID: 12468618. |
| Friis H, Gomo E, Nyazema N, Ndhlovu P, Krarup H, Madsen PH, Michaelsen KF. Iron, haptoglobin phenotype, and HIV-1 viral load: a cross-sectional study among pregnant Zimbabwean women. J Acquir Immune Defic Syndr. 2003 May 1;33(1):74-81. PubMed PMID: 12792358. |
| Garner P, Gelband H, Graves P, et al (2009) Systematic Reviews in Malaria: Global Policies Need Global Reviews. Infectious Disease Clinics of North America; 23 (2):387-404. |
| Gbotosho GO, Happi CT, Sijuade AO, Sowunmi A, Oduola AM. A simple cost-effective high performance liquid chromatographic assay of sulphadoxine in whole blood spotted on filter paper for field studies. Malar J. 2009 Oct 24;8:238. PubMed PMID: 19852850; PubMed Central PMCID: PMC2773786. |
| Gesase S, Gosling RD, Hashim R, et al (2009) High resistance of Plasmodium falciparum to sulphadoxine/pyrimethamine in Northern Tanzania and the emergence of dhps resistance mutation at codon 581. PLoS ONE; 4 (2): |
| Giha HA (2010) Prospects of intermittent preventive treatment of adults against malaria in areas of seasonal and unstable malaria transmission, and a possible role for chloroquine. Expert Opinion on Pharmacotherapy; 11 (6):871-876. |
| Gkrania-Klotsas E, Lever AML (2007) An update on malaria prevention, diagnosis and treatment for the returning traveller. Blood Reviews; 21 (2):73-87. |
| Gosling RD, Okell L, Mosha J, et al (2011) The role of antimalarial treatment in the elimination of malaria. Clinical Microbiology and Infection; 17 (11):1617-1623. |
| Graves PM, Gelband H, Ribeiro I (2009) Primaquine for reducing transmission of Plasmodium falciparum malaria Protocol. Cochrane Database of Systematic Reviews; 1: |
| Greenwood B (2004) The use of anti-malarial drugs to prevent malaria in the population of malaria-endemic areas. American Journal of Tropical Medicine and Hygiene; 70 (1):1-7. |
| Greenwood B (2006) Review: Intermittent preventive treatment - A new approach to the prevention of malaria in children in areas with seasonal malaria transmission. Tropical Medicine and International Health; 11 (7):983-991. |
| Greenwood B, Bojang K, Tagbor H, et al (2011) Combining community case management and intermittent preventive treatment for malaria. Trends in Parasitology; 27 (11):477-480. |
| Greenwood B. Progress in malaria control in endemic areas. Travel Med Infect Dis. 2008 Jul;6(4):173-6. Epub 2008 Jan 10. PubMed PMID: 18571103. |
| Greenwood BM (1997) What's new in malaria control? Annals of Tropical Medicine and Parasitology; 91 (5):523-531. |
| Griffith KS, Lewis LS, Mali S, et al (2007) Treatment of malaria in the United States: A systematic review. Journal of the American Medical Association; 297 (20):2264-2277. |
| Guerin PJ, Olliaro P, Nosten F, Druilhe P, Laxminarayan R, Binka F, Kilama WL, Ford N, White NJ. Malaria: current status of control, diagnosis, treatment, and a proposed agenda for research and development. Lancet Infect Dis. 2002 Sep;2(9):564-73. Review. PubMed PMID: 12206972. |
| Gutman J, Green M, Durand S, et al (2009) Mefloquine pharmacokinetics and mefloquine-artesunate effectiveness in Peruvian patients with uncomplicated Plasmodium falciparum malaria. Malaria journal; 8 (1): |
| Gutman J, Kachur SP. Treating malaria in pregnant women: a pressing problem. Lancet Infect Dis. 2010 Nov;10(11):739-40. PubMed PMID: 21029982. |
| Hahn S, Williamson PR, Hutton JL, et al (2000) Assessing the potential for bias in meta-analysis due to selective reporting of subgroup analyses within studies. Statistics in Medicine; 19 (24):3325-3336. |
| Hamel MJ, Poe A, Bloland P, et al (2008) Dihydrofolate reductase I164L mutations in Plasmodium falciparum isolates: clinical outcome of 14 Kenyan adults infected with parasites harbouring the I164L mutation. Transactions of the Royal Society of Tropical Medicine and Hygiene; 102 (4):338-345. |
| Harrington WE, Duffy PE (2008) Congenital malaria: Rare but potentially fatal. Pediatric Health; 2 (2):235-248. |
| Harte PG, Playfair JH. Failure of malaria vaccination in mice born to immune mothers. II. Induction of specific suppressor cells by maternal IgG. Clin Exp Immunol. 1983 Jan;51(1):157-64. PubMed PMID: 6219839; PubMed Central PMCID: PMC1536748. |
| Hartman TK, Rogerson SJ, Fischer PR (2010) The impact of maternal malaria on newborns. Annals of Tropical Paediatrics; 30 (4):271-282. |
| Hasan A, Parvez A, Shaheen, et al (2006) Pregnancy in patients with malaria. Journal, Indian Academy of Clinical Medicine; 7 (1):25-29. |
| Herrero MD, Rivas P, Rallon NI, et al (2007) HIV and malaria. AIDS Reviews; 9 (2):88-98. |
| Hewitt K, Steketee R, Mwapasa V, et al (2006) Interactions between HIV and malaria in non-pregnant adults: Evidence and implications. AIDS; 20 (16):1993-2004. |
| Hodel EM, Kabanywanyi AM, Malila A, Zanolari B, Mercier T, Beck HP, Buclin T, Olliaro P, Decosterd LA, Genton B. Residual antimalarials in malaria patients from Tanzania--implications on drug efficacy assessment and spread of parasite resistance. PLoS One. 2009 Dec 14;4(12):e8184. PubMed PMID: 20011529; PubMed Central PMCID: PMC2788605. |
| Hofheinz W, Bürgin H, Gocke E, Jaquet C, Masciadri R, Schmid G, Stohler H, Urwyler H. Ro 42-1611 (arteflene), a new effective antimalarial: chemical structure and biological activity. Trop Med Parasitol. 1994 Sep;45(3):261-5. PubMed PMID: 7899801. |
| Humphreys EH, Nageswaran A, Rutherford GW (2010) Artemisinin-based combination therapy for uncomplicated malaria in children with HIV Protocol. Cochrane Database of Systematic Reviews; 6: |
| Ibrahim ML, Steenkeste N, Khim N, et al (2009) Field-based evidence of fast and global increase of Plasmodium falciparum drug-resistance by DNA-microarrays and PCR/RFLP in Niger. Malaria journal; 8:32. |
| Idemyor V (2007) Human Immunodeficiency Virus (HIV) and malaria interaction in sub-Saharan Africa: The collision of two titans. HIV Clinical Trials; 8 (4):246-253. |
| Jamsen KM, Duffull SB, Tarning J, et al (2011) Optimal designs for population pharmacokinetic studies of oral artesunate in patients with uncomplicated falciparum malaria. Malaria journal; 10:181. |
| Jena J, Samal S, Rath B (2010) Reproductive profile of artemisinins in albino rats. Indian Journal of Pharmacology; 42 (3):192-193. |
| Jerrard DA, Broder JS, Hanna JR, et al (2002) Malaria: A rising incidence in the United States. Journal of Emergency Medicine; 23 (1):23-33. |
| Jha V, Chugh KS (2008) Community-Acquired Acute Kidney Injury in Asia. Seminars in Nephrology; 28 (4):330-347. |
| Jima D, Getachew A, Bilak H, Steketee RW, Emerson PM, Graves PM, Gebre T, Reithinger R, Hwang J; Ethiopia Malaria Indicator Survey Working Group. Malaria indicator survey 2007, Ethiopia: coverage and use of major malaria prevention and control interventions. Malar J. 2010 Feb 24;9:58. PubMed PMID: 20178654; PubMed Central PMCID: PMC2841196. |
| Jima D, Tesfaye G, Medhin A, et al (2005) Safety and efficacy of artemether-lumefantrine in the treatment of uncomplicated falciparum malaria in Ethiopia. East African Medical Journal; 82 (8):387-390. |
| Jimoh AAG. Recent trends in management of malaria in pregnancy. African Journal of Clinical and Experimental Microbiology 2006; 2:116-124 |
| Jones JS (2005) Malaria in Africa. South African Medical Journal; 95 (7):470-471. |
| Juckett RG (2005) Insect avoidance and malaria chemoprophylaxis. Clinics in Family Practice; 7 (4):653-673. |
| Kalilani L, Mofolo I, Chaponda M, et al (2007) A randomized controlled pilot trial of azithromycin or artesunate added to sulfadoxine-pyrimethamine as treatment for malaria in pregnant women. PLoS ONE; 2 (11):e1166. |
| Kalra SP, Naithani N, Mehta SR, et al (2002) Resistant malaria: Current concepts and therapeutic strategies. Medical Journal Armed Forces India; 58 (3):228-233. |
| Kamuhabwa A, Jalal R. Drug use in pregnancy: Knowledge of drug dispensers and pregnant women in Dar es Salaam, Tanzania. Indian J Pharmacol. 2011 May;43(3):345-9. PubMed PMID: 21713045; PubMed Central PMCID: PMC3113392. |
| Kamuhabwa,A.R., Mnyusiwalla,F. Rational dispensing and use of artemether-lumefantrine during pregnancy in Dar es Salaam, Tanzania. Tanzania Journal of Health Research, No 2 2011 |
| Kaye DK, Nshemerirwe R, Mutyaba TS, Ndeezi G. A randomized clinical trial comparing safety, clinical and parasitological response to artemether-lumefantrine and chlorproguanil-dapsone in treatment of uncomplicated malaria in pregnancy in Mulago hospital, Uganda. J Infect Dev Ctries. 2008 Apr 1;2(2):135-9. PubMed PMID: 19738339. |
| Kemble SK, Davis JC, Nalugwa T, et al (2006) Prevention and treatment strategies used for the community management of childhood fever in Kampala, Uganda. American Journal of Tropical Medicine and Hygiene; 74 (6):999-1007. |
| Kitua AY, Ogundahunsi OAT, Lines J, et al (2011) Conquering malaria: Enhancing the impact of effective interventions towards elimination in the diverse and changing epidemiology. Journal of Global Infectious Diseases; 3 (2):161-165. |
| Kondrachine AV, Trigg PI. Global overview of malaria. Indian J Med Res. 1997 Aug;106:39-52. Review. PubMed PMID: 9291675. |
| Krudsood S, Silachamroon U, Wilairatana P, et al (2000) A randomized clinical trial of combinations of artesunate and azithromycin for treatment of uncomplicated Plasmodium falciparum malaria in Thailand. The Southeast Asian journal of tropical medicine and public health; 31 (4):801-807. |
| Kurt TL (2008) Malaria prevention in short-term travelers. New England Journal of Medicine; 359 (21):2293-2294. |
| Kusel J, Hagan P (1999) Praziquantel - Its use, cost and possible development of resistance. Parasitology Today; 15 (9):352-354. |
| Lagerberg RE. Malaria in pregnancy: A literature review. Journal of Midwifery and women's health 2008;53(3):209-215 |
| Lalloo DG, Shingadia D, Pasvol G, Chiodini PL, Whitty CJ, Beeching NJ, Hill DR, Warrell DA, Bannister BA; HPA Advisory Committee on Malaria Prevention in UK ravellers. UK malaria treatment guidelines. J Infect. 2007 Feb;54(2):111-21. Epub 2007 Jan 9. PubMed PMID: 17215045. |
| Lee PW, Liu CT, do Rosario VE, de Sousa B, Rampao HS, Shaio MF. Potential threat of malaria epidemics in a low transmission area, as exemplified by São Tomé and Príncipe. Malar J. 2010 Sep 29;9:264. PubMed PMID: 20920216; PubMed Central PMCID: PMC2955676. |
| Lee PW, Liu CT, Rampao HS, do Rosario VE, Shaio MF. Pre-elimination of malaria on the island of Príncipe. Malar J. 2010 Jan 20;9:26. PubMed PMID: 20089158; PubMed Central PMCID: PMC2823607. |
| Lekana Douki JB, Traore B, Costa FT, Fusaï T, Pouvelle B, Sterkers Y, Scherf A, Gysin J. Sequestration of Plasmodium falciparum-infected erythrocytes to chondroitin sulfate A, a receptor for maternal malaria: monoclonal antibodies against the native parasite ligand reveal pan-reactive epitopes in placental isolates. Blood. 2002 Aug 15;100(4):1478-83. PubMed PMID: 12149234. |
| Li Q, Si Y, Smith KS, et al (2008) Embryotoxicity of artesunate in animal species related to drug tissue distribution and toxicokinetic profiles. Birth Defects Research Part B - Developmental and Reproductive Toxicology; 83 (4):435-445. |
| Li Q, Si Y, Xie L, et al (2009) Severe embryolethality of artesunate related to pharmacokinetics following intravenous and intramuscular doses in pregnant rats. Birth Defects Research; 86 (5):385-393. |
| Li Q, Weina P (2010) Artesunate: The best drug in the treatment of severe and complicated malaria. Pharmaceuticals; 3 (7):2322-2332. |
| Li Q, Weina PJ. Severe embryotoxicity of artemisinin derivatives in experimental animals, but possibly safe in pregnant women. Molecules. 2009 Dec 25;15(1):40-57. Review. PubMed PMID: 20110870. |
| Lindegardh N, Hanpithakpong W, Kamanikom B, Singhasivanon P, Socheat D, Yi P, Dondorp AM, McGready R, Nosten F, White NJ, Day NP. Major pitfalls in the measurement of artemisinin derivatives in plasma in clinical studies. J Chromatogr B Analyt Technol Biomed Life Sci. 2008 Dec 1;876(1):54-60. Epub 2008 Oct 18. PubMed PMID: 18980865. |
| Lockwood,C.J. How to stop a mother dying every minute. Contemporary OB/GY 2008; 11:12-15 |
| Longo M, Zanoncelli S, Della Torre P, Rosa F, Giusti A, Colombo P, Brughera M, Mazué G, Olliaro P. Investigations of the effects of the antimalarial drug dihydroartemisinin (DHA) using the Frog Embryo Teratogenesis Assay-Xenopus (FETAX). Reprod Toxicol. 2008 Aug;25(4):433-41. Epub 2008 Feb 29. PubMed PMID: 18394862. |
| Longo M, Zanoncelli S, Manera D, Brughera M, Colombo P, Lansen J, Mazué G, Gomes M, Taylor WR, Olliaro P. Effects of the antimalarial drug dihydroartemisinin (DHA) on rat embryos in vitro. Reprod Toxicol. 2006 Jan;21(1):83-93. Epub 2005 Jul 18. PubMed PMID: 16026965. |
| Longo M, Zanoncelli S, Torre PD, Riflettuto M, Cocco F, Pesenti M, Giusti A, Colombo P, Brughera M, Mazué G, Navaratman V, Gomes M, Olliaro P. In vivo and in vitro investigations of the effects of the antimalarial drug dihydroartemisinin (DHA) on rat embryos. Reprod Toxicol. 2006 Nov;22(4):797-810. Epub 2006 Aug 14. PubMed PMID: 16959470. |
| Lowe M (2000) Evidence-based medicine - The view from Fiji. Lancet; 356 (9235):1105-1107. |
| Lule JC, Wasswa J. Malaria - The obstetrician's dilemma: malaria in pregnancy: treatment problems. Uganda Medical Journal 1994; 2:7-10 |
| Lusingu JP, Lelo B, Mmbando BP, et al (2007) Use of preventive measures for malaria among women delivering in a rural district hospital in north-eastern Tanzania. American Journal of Tropical Medicine & Hygiene; 77 (5, Suppl. S):241-242. |
| Lusingu JP, Von SL (2008) Challenges in malaria control in sub-Saharan Africa: the vaccine perspective. Tanzania journal of health research; 10 (4):253-266. |
| Luzzi GA, Peto TE. Adverse effects of antimalarials. An update. Drug Saf. 1993 Apr;8(4):295-311. Review. PubMed PMID: 8481216. |
| Lynch C, Pearce R, Pota H, et al (2008) Emergence of a dhfr mutation conferring high-level drug resistance in Plasmodium falciparum populations from southwest Uganda. Journal of Infectious Diseases; 197 (11):1598-1604. |
| MacArthur JR, Kabanywanyi AM, Baja A, et al (2007) Efficacy of intermittent treatment with sulfadoxine-pyrimethamine alone or sulfadoxine-pyrimethamine plus artesunate for prevention of placental malaria in Tanzania. American Journal of Tropical Medicine & Hygiene; 77 (5, Suppl. S):238. |
| Mace KE, Lynch MF, MacArthur JR, et al (2011) Grand Rounds: The Opportunity for and Challenges to Malaria Eradication. Morbidity & Mortality Weekly Report; 60 (15):476-480. |
| Mahidol C (2004) Malaria: Integrated approaches for prevention and treatment. Acta Tropica; 89 (3):265-269. |
| malERA Consultative Group on Drugs (2011) A research agenda for malaria eradication: drugs. PLoS medicine; 8 (1):e1000402. |
| Mangham LJ, Cundill B, Ezeoke O, Nwala E, Uzochukwu BS, Wiseman V, Onwujekwe O. Treatment of uncomplicated malaria at public health facilities and medicine retailers in south-eastern Nigeria. Malar J. 2011 Jun 8;10:155. PubMed PMID: 21651787; PubMed Central PMCID: PMC3120734. |
| Manirakiza,A., Soula,G., Laganier,R., Klement,E., Djalle,D., Methode,M., Madji,N., Heredeibona,L.S., Le Faou,A., Delmont,J. Pattern of the antimalarials prescription during pregnancy in Bangui, Central African Republic. Malaria Research and Treatment 2011;2011, article ID 414510 |
| Manyando C, Mkandawire R, Puma L, Sinkala M, Mpabalwani E, Njunju E, Gomes M, Ribeiro I, Walter V, Virtanen M, Schlienger R, Cousin M, Chipimo M, Sullivan FM. Safety of artemether-lumefantrine in pregnant women with malaria: results of a prospective cohort study in Zambia. Malar J. 2010 Sep 1;9:249. PubMed PMID: 20809964; PubMed Central PMCID: PMC2944339. |
| Masanja MI, McMorrow M, Kahigwa E, Kachur SP, McElroy PD. Health workers' use of malaria rapid diagnostic tests (RDTs) to guide clinical decision making in rural dispensaries, Tanzania. Am J Trop Med Hyg. 2010 Dec;83(6):1238-41. PubMed PMID: 21118927; PubMed Central PMCID: PMC2990037. |
| Mathanga DP, Uthman OA, Chinkhumba J (2011) Intermittent preventive treatment regimens for malaria in HIV-positive pregnant women Systematic Review. Cochrane Database of Systematic Reviews; 10: |
| McClure EM, Saleem S, Pasha O, et al (2009) Stillbirth in developing countries: A review of causes, risk factors and prevention strategies. Journal of Maternal-Fetal and Neonatal Medicine; 22 (3):183-190. |
| McGready R, Ashley EA, Moo E, Cho T, Barends M, Hutagalung R, Looareesuwan S, White NJ, Nosten F. A randomized comparison of artesunate-atovaquone-proguanil versus quinine in treatment for uncomplicated falciparum malaria during pregnancy. J Infect Dis 2005;192(5):846-853 |
| McGready R, Ashley EA, Nosten F (2004) Malaria and the pregnant traveller. Travel Medicine and Infectious Disease; 2 (3-4):127-142. |
| McGready R, Ashley EA, Tan SO, et al (2006) Re: Malaria in pregnancy 3. BJOG: An International Journal of Obstetrics and Gynaecology; 113 (2):246. |
| McGready R, Ashley EA, Wuthiekanun V, Tan SO, Pimanpanarak M, Viladpai-Nguen SJ, Jesadapanpong W, Blacksell SD, Peacock SJ, Paris DH, Day NP, Singhasivanon P, White NJ, Nosten F. Arthropod borne disease: the leading cause of fever in pregnancy on the Thai-Burmese border. PLoS Negl Trop Dis. 2010 Nov 16;4(11):e888. PubMed PMID: 21103369; PubMed Central PMCID: PMC2982829. |
| McGready R, Brockman A, Cho T, Cho D, van Vugt M, Luxemburger C, Chongsuphajaisiddhi T, White NJ, Nosten F. Randomized comparison of mefloquine-artesunate versus quinine in the treatment of multidrug-resistant falciparum malaria in pregnancy. Trans Royal Soc Trop Med Hyg 2000;94(6):689-693 |
| McGready R, Cho T, Cho JJ, Simpson JA, Luxemburger C, Dubowitz L, Looareesuwan S, White NJ, Nosten F. Artemisinin derivatives in the treatment of falciparum malaria in pregnancy. Trans R Soc Trop Med Hyg. 1998 Jul-Aug;92(4):430-3. PubMed PMID: 9850401. |
| McGready R, Cho T, Keo NK, Thwai KL, Villegas L, Looareesuwan S, White NJ, Nosten F. Artemisinin antimalarials in pregnancy: a prospective treatment study of 539 episodes of multidrug-resistant Plasmodium falciparum. Clin Infect Dis. 2001 Dec 15;33(12):2009-16. Epub 2001 Nov 9. PubMed PMID: 11712093. |
| McGready R, Cho T, Samuel, et al (2001) Randomized comparison of quinine-clindamycin versus artesunate in the treatment of falciparum malaria in pregnancy. Transactions of the Royal Society of Tropical Medicine and Hygiene; 95 (6):651-656. |
| McGready R, Keo NK, Villegas L, et al (2003) Artesunate-atovaquone-proguanil rescue treatment of multidrug-resistant Plasmodium falciparum malaria in pregnancy: a preliminary report. Transactions of the Royal Society of Tropical Medicine & Hygiene; 97 (5):592-594. |
| McGready R, Nosten F (1999) The Thai-Burmese border: drug studies of Plasmodium falciparum in pregnancy. Annals of Tropical Medicine & Parasitology; 93 Suppl 1:S19-S23. |
| McGready R, Nosten F. Which drug is effective and safe for acute malaria in pregnancy? Reviewing the evidence. Drug Development Research 2009;71(1):56-68 |
| McGready R, Stepniewska K, Edstein MD, et al (2003) The pharmacokinetics of atovaquone and proguanil in pregnant women with acute falciparum malaria. European Journal of Clinical Pharmacology; 59 (7):545-552. |
| McGready R, Stepniewska K, Lindegardh N, Ashley EA, La Y, Singhasivanon P,White NJ, Nosten F. The pharmacokinetics of artemether and lumefantrine in pregnant women with uncomplicated falciparum malaria. Eur J Clin Pharmacol. 2006 Dec;62(12):1021-31. Epub 2006 Oct 20. Erratum in: Eur J Clin Pharmacol. 2009 Aug;65(8):847. PubMed PMID: 17053895. |
| McGready R, Stepniewska K, Ward SA, et al (2006) Pharmacokinetics of dihydroartemisinin following oral artesunate treatment of pregnant women with acute uncomplicated falciparum malaria. European Journal of Clinical Pharmacology; 62 (5):367-371. |
| McGready R, Tan SO, Ashley EA, Pimanpanarak M, Viladpai-Nguen J, Phaiphun L, Wüstefeld K, Barends M, Laochan N, Keereecharoen L, Lindegardh N, Singhasivanon P, White NJ, Nosten F. A randomised controlled trial of artemether-lumefantrine versus artesunate for uncomplicated plasmodium falciparum treatment in pregnancy. PLoS Med. 2008 Dec 23;5(12):e253. PubMed PMID: 19265453; PubMed Central PMCID: PMC2605900. |
| McGready R, White NJ, Nosten F. Parasitological efficacy of antimalarials in the treatment and prevention of falciparum malaria in pregnancy 1998 to 2009: a systematic review. BJOG. 2011 Jan;118(2):123-35. doi: 10.1111/j.1471-0528.2010.02810.x. Review. PubMed PMID: 21159117. |
| McIntosh H, Jones KL (2009) Chloroquine or amodiaquine combined with sulfadoxine-pyrimethamine for treating uncomplicated malaria Systematic Review. Cochrane Database of Systematic Reviews; 1: |
| McIntosh H, Olliaro P (2009) Artemisinin derivatives for treating uncomplicated malaria Systematic Review. Cochrane Database of Systematic Reviews; 1: |
| Medhi B, Patyar S, Rao RS, Byrav D S P, Prakash A. Pharmacokinetic and toxicological profile of artemisinin compounds: an update. Pharmacology. 2009;84(6):323-32. Epub 2009 Oct 23. Review. PubMed PMID: 19851082. |
| Mehta SR, Das S. Management of malaria: recent trends. J Commun Dis. 2006 Mar;38(2):130-8. Review. PubMed PMID: 17370675. |
| Menendez C, D'Alessandro U, ter Kuile F. Reducing the burden of malaria in pregnancy by preventive strategies. The Lancet Infectious Diseases 2007;7(2):126-135 |
| Menendez C, Mayor A (2007) Congenital malaria: The least known consequence of malaria in pregnancy. Seminars in Fetal and Neonatal Medicine; 12 (3):207-213. |
| Mens PF, van Overmeir C, Bonnet M, Dujardin JC, d'Alessandro U. Real-time PCR/MCA assay using fluorescence resonance energy transfer for the genotyping of resistance related DHPS-540 mutations in Plasmodium falciparum. Malar J. 2008 Mar 17;7:48. PubMed PMID: 18346279; PubMed Central PMCID: PMC2276220. |
| Meremikwu MM, Donegan S, Esu E (2009) Chemoprophylaxis and intermittent treatment for preventing malaria in children Systematic Review. Cochrane Database of Systematic Reviews; 1: |
| Milner DA Jr, Montgomery J, Seydel KB, Rogerson SJ. Severe malaria in children and pregnancy: an update and perspective. Trends Parasitol. 2008 Dec;24(12):590-5. Epub 2008 Oct 8. Review. PubMed PMID: 18848498. |
| Mimche PN, Taramelli D, Vivas L (2011) The plant-based immunomodulator curcumin as a potential candidate for the development of an adjunctive therapy for cerebral malaria. Malaria Journal; 10 (SUPPL. 1): |
| Miranda ES, Osorio-de-Castro CGS (2009) Control policies and interventions focusing on malaria in pregnancy. Current Women's Health Reviews; 5 (4):239-246. |
| Mirghani RA, Elagib I, Elghazali G, Hellgren U, Gustafsson LL. Effects of Plasmodium falciparum infection on the pharmacokinetics of quinine and its metabolites in pregnant and non-pregnant Sudanese women. Eur J Clin Pharmacol. 2010 Dec;66(12):1229-34. Epub 2010 Aug 18. PubMed PMID: 20717655. |
| Mirghani RA, Elagib I, Hellgren U, et al (2003) Activity of CYP3A determined by quinine hydroxylation in pregnant and non pregnant women infected with Plasmodium falciparum. Tulunay, F; C:Towards. |
| Mishra DN (1998) Malaria in pregnancy. Journal of Internal Medicine of India; 1 (1):69-71. |
| Mishra SK, Mohanty S, Mohanty A, et al (2006) Management of severe and complicated malaria. Journal of Postgraduate Medicine; 52 (4):281-287. |
| Mishra SK, Satpathy R, Panigrahi P (2007) Malaria awareness of village healthworkers from Sundargarh in Orissa - The state contributing half of themalaria-related deaths in India. Tropical Doctor; 37 (2):126-127. |
| Mkulama MA, Chishimba S, Sikalima J, Rouse P, Thuma PE, Mharakurwa S. Escalating Plasmodium falciparum antifolate drug resistance mutations in Macha, rural Zambia. Malar J. 2008 May 21;7:87. PubMed PMID: 18495008; PubMed Central PMCID: PMC2412897. |
| Mohan A, Sharma SK, Bollineni S (2008) Acute lung injury and acute respiratory distress syndrome in malaria. Journal of Vector Borne Diseases; 45 (3):179-193. |
| Morel CM (2006) Achieving the millennium development goals for health: Cost effectiveness analysis of strategies to combat malaria in developing countries (vol 331, pg 1299, 2005). BMJ; 333 (7558):86. |
| Morel CM, Lauer JA, Evans DB (2005) Achieving the millennium development goals for health: Cost effectiveness analysis of strategies to combat malaria in developing countries. British Medical Journal; 331 (7528):1299-1302. |
| Morel CM, Lauer JA, Evans DB (2005) Cost effectiveness analysis of strategies to combat malaria in developing countries. BMJ (Clinical research ed.); 331 (7528):1299. |
| Morris CA, Duparc S, Borghini-Fuhrer I, Jung D, Shin CS, Fleckenstein L. Review of the clinical pharmacokinetics of artesunate and its active metabolite dihydroartemisinin following intravenous, intramuscular, oral or rectal administration. Malar J. 2011 Sep 13;10:263. PubMed PMID: 21914160; PubMed Central PMCID: PMC3180444. |
| Morris CA, Onyamboko MA, Capparelli E, Koch MA, Atibu J, Lokomba V, Douoguih M, Hemingway-Foday J, Wesche D, Ryder RW, Bose C, Wright L, Tshefu AK, Meshnick S, Fleckenstein L. Population pharmacokinetics of artesunate and dihydroartemisinin in pregnant and non-pregnant women with malaria. Malar J. 2011 May 8;10:114. PubMed PMID: 21548983; PubMed Central PMCID: PMC3098207. |
| Mturi N, Musumba CO, Wamola BM, Ogutu BR, Newton CR. Cerebral malaria: optimising management. CNS Drugs. 2003;17(3):153-65. Review. PubMed PMID: 12617695. |
| Mubyazi GM, Bygbjerg IC, Magnussen P, et al (2008) Prospects, achievements, challenges and opportunities for scaling-up malaria chemoprevention in pregnancy in Tanzania: The perspective of national level officers. Malaria journal; 7: |
| Mulenga M, VangGeertruyden JP, Mwananyanda L, et al Safety and efficacy of lumefantrine-artemether (Coartem) for the treatment of uncomplicated Plasmodium falciparum malaria in Zambian adults. Malaria journal; Vol.5, pp.73, 2006.:73. |
| Mutabingwa TK, Muze K, Ord R, Briceno M, Greenwood BM, Drakelyey C, Whitty CJM. Randomized Trial of Artesunate+Amodiaquine, Sulfadoxine-Pyrimethamine+Amodiaquine, Chlorproguanal-Dapsone and SP for Malaria in Pregnancy in Tanzania. PloS ONE 2008; 4(4) e5138. doi:10.1371/journal.pone.0005138 |
| Mutabingwa TK. Artemisinin-based combination therapies (ACTs): best hope for malaria treatment but inaccessible to the needy! Acta Trop. 2005 Sep;95(3):305-15. Review. PubMed PMID: 16098946. |
| Mutabingwa TK. Treating malaria during pregnancy in Africa. Postgraduate Doctor Africa 2003;4 |
| Myint HY, Tipmanee P, Nosten F, et al (2004) A systematic overview of published antimalarial drug trials. Transactions of the Royal Society of Tropical Medicine and Hygiene; 98 (2):73-81. |
| Na-Bangchang K (2009) Pharmacodynamics of antimalarial chemotherapy. Expert Review of Clinical Pharmacology; 2 (5):491-515. |
| Na-Bangchang K, Congpuong K (2007) Current malaria status and distribution of drug resistance in East and Southeast Asia with special focus to Thailand. Tohoku Journal of Experimental Medicine; 211 (2):99-113. |
| Nahum A, Erhart A, Gazard D, et al (2007) Adding artesunate to sulphadoxine-pyrimethamine greatly improves the treatment efficacy in children with uncomplicated falciparum malaria on the coast of Benin, West Africa. Malaria journal; 6: |
| Nambozi M, Van Geertruyden JP, Hachizovu S, et al (2011) Safety and efficacy of dihydroartemisinin-piperaquine versus artemether-lumefantrine in the treatment of uncomplicated Plasmodium falciparum malaria in Zambian children. Malaria journal; 10:50. |
| Ndiaye JL, Randrianarivelojosia M, Sagara I, Brasseur P, Ndiaye I, Faye B, Randrianasolo L, Ratsimbasoa A, Forlemu D, Moor VA, Traore A, Dicko Y, Dara N, Lameyre V, Diallo M, Djimde A, Same-Ekobo A, Gaye O. Randomized, multicentre assessment of the efficacy and safety of ASAQ--a fixed-dose artesunate-amodiaquine combination therapy in the treatment of uncomplicated Plasmodium falciparum malaria. Malar J. 2009 Jun 8;8:125. PubMed PMID: 19505304; PubMed Central PMCID: PMC2698916. |
| Newman RD, Parise ME, Slutsker L, Nahlen B, Steketee RW. Safety, efficacy and determinants of effectiveness of antimalarial drugs during pregnancy: implications for prevention programmes in Plasmodium falciparum-endemic sub-Saharan Africa. Trop Med Int Health. 2003 Jun;8(6):488-506. Review. PubMed PMID: 12791054. |
| Newton PN, Hampton CY, Alter-Hall K, et al (2008) Characterization of "Yaa Chud" medicine on the Thailand-Myanmar border: Selecting for drug-resistant malaria and threatening public health. American Journal of Tropical Medicine and Hygiene; 79 (5):662-669. |
| Nilkaeo A, Bhuvanath S, Praputbut S, Wisessombat S. Induction of cell cycle arrest and apoptosis in JAR trophoblast by antimalarial drugs. Biomed Res. 2006 Jun;27(3):131-7. PubMed PMID: 16847359. |
| Niu XY, Ho LY, Ren ZH, Song ZY. Metabolic fate of Qinghaosu in rats; a new TLC densitometric method for its determination in biological material. Eur J Drug Metab Pharmacokinet. 1985 Jan-Mar;10(1):55-9. PubMed PMID: 4029221. |
| Njuguna P, Newton C. Management of severe falciparum malaria. J Postgrad Med. 2004 Jan-Mar;50(1):45-50. PubMed PMID: 15047999. |
| Noedl H (2009) ABC - antibiotics-based combinations for the treatment of severe malaria? Trends in Parasitology; 25 (12):540-544. |
| Noedl H, Krudsood S, Chalermratana K, et al (2006) Azithromycin combination therapy with artesunate or quinine for the treatment of uncomplicated Plasmodium falciparum malaria in adults: a randomized, phase 2 clinical trial in Thailand. Clinical infectious diseases : an official publication of the Infectious Diseases Society of America; 43 (10):1264-1271. |
| Noedl H, Wernsdorfer WH, Krudsood S, et al (2001) Antimalarial activity of azithromycin, artemisinin and dihydroartemisinin in fresh isolates of Plasmodium falciparum in Thailand. Acta Tropica; 80 (1):39-44. |
| Nosten F, Ashley E, McGready R, et al (2006) We still need artesunate monotherapy 4. British Medical Journal; 333 (7557):45. |
| Nosten F, McGready R, Ashley E, et al (2005) Malaria misconceptions 3. Lancet; 365 (9460):653. |
| Nosten F, McGready R, d'Alessandro U, Bonell A, Verhoeff F, Menendez C, Mutabingwa T, Brabin B. Antimalarial drugs in pregnancy: a review. Curr Drug Saf. 2006 Jan;1(1):1-15. Review. PubMed PMID: 18690910. |
| Nosten F, McGready R, Mutabingwa T. Case management of malaria in pregnancy. The Lancet Infectious Diseases 2007;7(2):118-125 |
| Nosten F, Price RN. New antimalarials. A risk-benefit analysis. Drug Saf. 1995 Apr;12(4):264-73. Review. PubMed PMID: 7646825. |
| Nosten F, Rogerson SJ, Beeson JG, McGready R, Mutabingwa TK, Brabin B. Malaria in pregnancy and the endemicity spectrum: what can we learn? Trends in Parasitology 2004;20(9):425-432 |
| Nosten F, van Vugt M, Price R, Luxemburger C, Thway KL, Brockman A, McGready R, ter Kuile F, Looareesuwan S, White NJ. Effects of artesunate-mefloquine combination on incidence of Plasmodium falciparum malaria and mefloquine resistance in western Thailand: a prospective study. Lancet. 2000 Jul 22;356(9226):297-302. PubMed PMID: 11071185. |
| Nosten F, White NJ. Artemisinin-based combination treatment of falciparum malaria. Am J Trop Med Hyg. 2007 Dec;77(6 Suppl):181-92. Review. PubMed PMID: 18165491. |
| Nsimba B, Guiyedi V, Mabika-Mamfoumbi M, et al (2008) Sulphadoxine/pyrimethamine versus amodiaquine for treating uncomplicated childhood malaria in Gabon: a randomized trial to guide national policy. Malaria journal; 7:31. |
| Nsimba SE (2011) Antimalarial drug combination chemotherapy in malaria case management in Tanzania: How did it come about? Therapy; 8 (5):563-568. |
| Nyunt MM, Plowe CV. Pharmacologic advances in the global control and treatment of malaria: combination therapy and resistance. Clin Pharmacol Ther. 2007 Nov;82(5):601-5. Epub 2007 Sep 26. PubMed PMID: 17898709. |
| Ochong E, Bell DJ, Johnson DJ, D'Alessandro U, Mulenga M, Muangnoicharoen S, Van Geertruyden JP, Winstanley PA, Bray PG, Ward SA, Owen A. Plasmodium falciparum strains harboring dihydrofolate reductase with the I164L mutation are absent in Malawi and Zambia even under antifolate drug pressure. Antimicrob Agents Chemother. 2008 Nov;52(11):3883-8. Epub 2008 Aug 25. PubMed PMID: 18725445; PubMed Central PMCID: PMC2573156. |
| Ogbonna A, Uneke CJ. Artemisinin-based combination therapy for uncomplicated malaria in sub-Saharan Africa: the efficacy, safety, resistance and policy implementation since Abuja 2000. Trans R Soc Trop Med Hyg. 2008 Jul;102(7):621-7. Epub 2008 May 21. Review. PubMed PMID: 18499204. |
| Ogunwande SA. Study of malarial chemoprophylaxis and pregnancy gingivitis in Nigerian women. Clin Prev Dent. 1991 Sep-Oct;13(5):25-30. PubMed PMID: 1809526. |
| Oh S, Jeong IH, Shin WS, Lee S. Growth inhibition activity of thioacetal artemisinin derivatives against human umbilical vein endothelial cells. Bioorg Med Chem Lett. 2003 Nov 3;13(21):3665-8. PubMed PMID: 14552753. |
| Okogun GRA, Amadi AN (2005) Epidemiology, therapeutic agents and cost of management of paediatric malaria in a Nigerian tertiary hospital. Journal of Vector Borne Diseases; 42 (3):87-94. |
| Okpere,E.E., Enabudoso,E.J., Osemwenkha,A.P. Malaria in pregnancy. Nigerian Medical Journal 2010;3:109-113 |
| Okunlola MA, Akinwuntan AL, Fehintola FA (2006) Cerebral malaria in pregnancy. Journal of Obstetrics and Gynaecology; 26 (6):561. |
| Ologe OM, Mokuolu OA, Adedoyin OT (2007) Pattern of antimalarial prescriptions for children and pregnant mothers by private medical practitioners in Ilorin, Nigeria. Tropical Doctor; 37 (4):212-214. |
| Omari AA, Garner P (2007) Malaria: severe, life-threatening. Clinical Evidence; 2007, 2007.: |
| Omo-Aghoja LO, Abe E, Feyi-Waboso P, et al (2008) The challenges of diagnosis and treatment of malaria in pregnancy in low resource settings. Acta Obstetricia et Gynecologica Scandinavica; 87 (7):693-696. |
| Omole,M.K., Onwusah,O.D. A ten year study of the treatment of malaria in pregnancy at a secondary hospital in south west Nigeria. International Journal of Pharmaceutical and Biomedical Research 2011. Vol 1; 22-25 |
| Ondimu KN. Determinants of perinatal health problems in Kisumu district, Kenya. International Journal of Health Care Quality Assurance 2001;5:200-211 |
| Onyamboko MA, Meshnick SR, Fleckenstein L, Koch MA, Atibu J, Lokomba V,Douoguih M, Hemingway-Foday J, Wesche D, Ryder RW, Bose C, Wright LL, Tshefu AK, Capparelli EV. Pharmacokinetics and pharmacodynamics of artesunate and dihydroartemisinin following oral treatment in pregnant women with asymptomatic Plasmodium falciparum infections in Kinshasa DRC. Malar J. 2011 Feb 28;10:49. PubMed PMID: 21352601; PubMed Central PMCID: PMC3056842. |
| Orton LC, Omari AAA (2008) Drugs for treating uncomplicated malaria in pregnant women. Cochrane Database of Systematic Reviews; (4): |
| OseiAkoto A, Orton LC, OwusuOfori S (2011) Atovaquone-proguanil for treating uncomplicated malaria Systematic Review. Cochrane Database of Systematic Reviews; 1: |
| Ovid references |
| Petersen E (2004) Malaria chemoprophylaxis: When should we use it and what are the options? Expert Review of Anti-Infective Therapy; 2 (1):119-132. |
| Phillips RS (2001) Current status of malaria and potential for control. Clinical Microbiology Reviews; 14 (1):208-226. |
| Phillips-Howard PA, Wood D. The safety of antimalarial drugs in pregnancy.Drug Saf. 1996 Mar;14(3):131-45. Review. PubMed PMID: 8934576. |
| Piola P, Fogg C, Bajunirwe F, et al (2005) Supervised versus unsupervised intake of six-dose artemether-lumefantrine for treatment of acute, uncomplicated Plasmodium falciparum malaria in Mbarara, Uganda: A randomised trial. Lancet; 365 (9469):1467-1473. |
| Piola P, Nabasumba C, Turyakira E, Dhorda M, Lindegardh N, Nyehangane D, Snounou G, Ashley EA, McGready R, Nosten F, Guerin PJ. Efficacy and safety of artemether-lumefantrine compared with quinine in pregnant women with uncomplicated Plasmodium falciparum malaria: an open-label, randomised, non-inferiority trial. Lancet Infect Dis. 2010 Nov;10(11):762-9. PubMed PMID: 20932805. |
| Plowe CV, Roper C, Barnwell JW, et al (2007) World Antimalarial Resistance Network (WARN) III: Molecular markers for drug resistant malaria. Malaria Journal; 6: 121 |
| Poespoprodjo JR, Fobia W, Kenangalem E, et al (2008) Adverse pregnancy outcomes in an area where multidrug-resistant plasmodium vivax and Plasmodium falciparum infections are endemic. Clinical Infectious Diseases; 46 (9):1374-1381. |
| Poespoprodjo JR, Fobia W, Kenangalem E, Hasanuddin A, Sugiarto P, Tjitra E, Anstey NM, Price RN. Highly effective therapy for maternal malaria associated with a lower risk of vertical transmission. J Infect Dis. 2011 Nov;204(10):1613-9. Epub 2011 Sep 9. PubMed PMID: 21908728. |
| Poespoprodjo JR, Hasanuddin A, Fobia W, et al (2010) Severe congenital malaria acquired in utero. The American journal of tropical medicine and hygiene; 82 (4):563-565. |
| Pogun S, Yararbas G (2009) Sex differences in nicotine action. Nicotine Psychopharmacology; Handbook of Experimental Pharmacology. 192:261-291. |
| Poirot E, Hwang J, Kachur PS, et al (2010) Mass drug administration for malaria Protocol. Cochrane Database of Systematic Reviews; 11: |
| Ponsar F, Van HM, Zachariah R, et al (2011) Abolishing user fees for children and pregnant women trebled uptake of malaria-related interventions in Kangaba, Mali. Health Policy & Planning; 26 Suppl 2:ii72-ii83. |
| Pottie K, Greenaway C, Feightner J, et al (2011) Evidence-based clinical guidelines for immigrants and refugees. CMAJ; 183 (12):E824-E925. |
| Pukrittayakamee S, White NJ (2001) Combination therapy: Making the best use of existing drugs. Pharmaceutical News; 8 (5):21-26. |
| Ratakonda U (1998) Malaria complicating pregnancy: Report of two cases and review of management. Primary Care Update for Ob/Gyns; 5 (6):306-310. |
| Rath B, Jena J, Samal S, et al (2010) Reproductive profile of artemisinins in albino rats. Indian Journal of Pharmacology; 42 (3):192. |
| Rathore D, McCutchan TF, Sullivan M, et al (2005) Antimalarial drugs: Current status and new developments. Expert Opinion on Investigational Drugs; 14 (7):871-883. |
| Rijken MJ, McGready R, Boel ME, Barends M, Proux S, Pimanpanarak M, Singhasivanon P, Nosten F. Dihydroartemisinin-piperaquine rescue treatment of multidrug-resistant Plasmodium falciparum malaria in pregnancy: a preliminary report. Am J Trop Med Hyg. 2008 Apr;78(4):543-5. PubMed PMID: 18385345. |
| Rijken MJ, McGready R, Phyo AP, Lindegardh N, Tarning J, Laochan N, Than HH, Mu O, Win AK, Singhasivanon P, White N, Nosten F. The pharmacokinetics of dihydroartemisinin and piperaquine in pregnant and non-pregnant women with uncomplicated falciparum malaria. Antimicrob Agents Chemother. 2011 55 (12):5500-5506. PubMed PMID: 21947392. |
| Ringsted FM, Massawe IS, Lemnge MM, Bygbjerg IC. Saleability of anti-malarials in private drug shops in Muheza, Tanzania: a baseline study in an era of assumed artemisinin combination therapy (ACT). Malar J. 2011 Aug 15;10:238. PubMed PMID: 21843328; PubMed Central PMCID: PMC3167767. |
| Riscoe M, Kelly JX, Winter R (2005) Xanthones as antimalarial agents: Discovery, mode of action, and optimization. Current Medicinal Chemistry; 12 (21):2539-2549. |
| Rogerson SJ (2010) Malaria in pregnancy and the newborn. Hot Topics in Infection and Immunity in Children VI; Advances in Experimental Medicine and Biology. 659:139-152. |
| Rogerson SJ, Menendez C. Treatment and prevention of malaria in pregnancy: opportunities and challenges. Expert Review of Anti-Infective Therapy 2006;4(4):687-702 |
| Rogerson SJ, Wijesinghe RS, Meshnick SR (2010) Host immunity as a determinant of treatment outcome in Plasmodium falciparum malaria. The Lancet Infectious Diseases; 10 (1):51-59. |
| Rolland E, Checchi F, Pinoges L, Balkan S, Guthmann JP, Guerin PJ.Operational response to malaria epidemics: are rapid diagnostic tests cost-effective? Trop Med Int Health. 2006 Apr;11(4):398-408. PubMed PMID:16553923. |
| Rollinson D (2009) A wake up call for urinary schistosomiasis: Reconciling research effort with public health importance. Parasitology; 136 (12):1593-1610. |
| Rosenblatt JE. Antiparasitic agents. Mayo Clin Proc. 1999 Nov;74(11):1161-75. Review. PubMed PMID: 10560606. |
| Sagara I, Rulisa S, Mbacham W, Adam I, Sissoko K, Maiga H, Traore OB, Dara N, Dicko YT, Dicko A, Djimdé A, Jansen FH, Doumbo OK. Efficacy and safety of a fixed dose artesunate-sulphamethoxypyrazine-pyrimethamine compared to artemether-lumefantrine for the treatment of uncomplicated falciparum malaria across Africa: a randomized multi-centre trial. Malar J. 2009 Apr 14;8:63. PubMed PMID: 19366448; PubMed Central PMCID: PMC2678145. |
| Sangaré LR, Weiss NS, Brentlinger PE, Richardson BA, Staedke SG, Kiwuwa MS, Stergachis A. Patterns of anti-malarial drug treatment among pregnant women in Uganda. Malar J. 2011 Jun 6;10:152. PubMed PMID: 21645402; PubMed Central PMCID: PMC3118160. |
| Sartelet H, Garraud O, Rogier C, Milko-Sartelet I, Kaboret Y, Michel G, Roussilhon C, Huerre M, Gaillard D. Hyperexpression of ICAM-1 and CD36 in placentas infected with Plasmodium falciparum: a possible role of these molecules in sequestration of infected red blood cells in placentas. Histopathology. 2000 Jan;36(1):62-8. PubMed PMID: 10632754. |
| Sayang C, Gausseres M, Vernazza-Licht N, Malvy D, Bley D, Millet P. Treatment of malaria from monotherapy to artemisinin-based combination therapy by health professionals in urban health facilities in Yaoundé, central province, Cameroon. Malar J. 2009 Jul 29;8:176. PubMed PMID: 19640292; PubMed Central PMCID: PMC2726182. |
| Sayang C, Gausseres M, Vernazza-Licht N, Malvy D, Bley D, Millet P. Treatment of malaria from monotherapy to artemisinin-based combination therapy by health professionals in urban health facilities in Yaoundé, central province, Cameroon. Malar J. 2009 Jul 29;8:176. PubMed PMID: 19640292; PubMed Central PMCID: PMC2726182. |
| Schellenberg D, Abdulla S, Roper C. Current issues for anti-malarial drugs to control P. falciparum malaria. Curr Mol Med. 2006 Mar;6(2):253-60. Review. PubMed PMID: 16515515. |
| Schlagenhauf P, Petersen E (2008) Malaria chemoprophylaxis: Strategies for risk groups. Clinical Microbiology Reviews; 21 (3):466-472. |
| Schmuck G, Klaus AM, Krotlinger F, et al (2009) Developmental and reproductive toxicity studies on artemisone. Birth Defects Research; 86 (2):131-143. |
| Seal SL, Mukhopadhay S, Ganguly RP (2010) Malaria in pregnancy. Journal of the Indian Medical Association; 108 (8):487-490. |
| Sevene E, Gonzalez R, Menendez C. Current knowledge and challenges of antimalarial drugs for treatment and prevention in pregnancy. Expert Opinion on Pharmacotherapy 2010;11(8):1277-1293 |
| Shanks GD, Edstein MD (2005) Modern malaria chemoprophylaxis. Drugs; 65 (15):2091-2110. |
| Sharma VP (2009) Hidden burden of malaria in Indian women. Malaria journal; 8 (1): |
| Sheehy SH, Angus BJ (2011) Malaria: severe, life-threatening. Clinical Evidence; 2011, 2011.: |
| Shulman CE, Dorman EK. Importance and prevention of malaria in pregnancy. Trans Royal Soc Trop Med Hyg 2003;97(1):30-35 |
| Sierra LA (2005) Evidence basis for antimalarial policy change in Sierra Leone: five in vivo efficacy studies of chloroquine, sulphadoxine-pyrimethamine and amodiaquine. Tropical Medicine & International Health; 10 (2):146-153. |
| Silver HM (1997) Malarial infection during pregnancy. Infectious Disease Clinics of North America; 11 (1):99-107. |
| Sinclair D, Donegan S, Lalloo DG (2010) Artesunate versus quinine for treating severe malaria Systematic Review. Cochrane Database of Systematic Reviews; 8: |
| Sinclair D, Gogtay N, Brand F, et al (2010) Artemisinin-based combination therapy for treating uncomplicated malaria Systematic Review. Cochrane Database of Systematic Reviews; 8: |
| Sinclair D, Zani B, Donegan S, et al (2009) Artemisinin-based combination therapy for treating uncomplicated malaria Systematic Review. Cochrane Database of Systematic Reviews; 1: |
| Sirima SB, Gansane A (2007) Artesunate-amodiaquine for the treatment of uncomplicated malaria. Expert Opinion on Investigational Drugs; 16 (7):1079-1085. |
| Slutsker L, Marston BJ (2007) HIV and malaria: Interactions and implications. Current Opinion in Infectious Diseases; 20 (1):3-10. |
| Smereck J. Malaria in pregnancy: update on emergency management. The Journal of Emergency Medicine 2011;40(4):393-396 |
| Southgate VR, Rollinson D, Tchuem Tchuente LA, et al (2005) Towards control of schistosomiasis in sub-Saharan Africa. Journal of Helminthology; 79 (3 SPEC. ISS.):181-185. |
| Sowunmi A, Oduola AM, Ogundahunsi OA, Fehintola FA, Ilesanmi OA, Akinyinka OO, Arowojolu AO. Randomised trial of artemether versus artemether and mefloquine for the treatment of chloroquine/sufadoxine-pyrimethamine-resistant falciparum malaria during pregnancy. J Obstet Gynaecol. 1998 Jul;18(4):322-7. PubMed PMID: 15512100. |
| Stanisic DI, Martin LB, Good MF. The role of the 19-kDa region of merozoite surface protein 1 and whole-parasite-specific maternal antibodies in directing neonatal pups' responses to rodent malaria infection. J Immunol. 2003 Nov 15;171(10):5461-9. PubMed PMID: 14607952. |
| Stanley J (1997) Malaria. Emergency Medicine Clinics of North America; 15 (1):113-156. |
| Steketee RW (2009) Good News in Malaria Control ... Now What? American Journal of Tropical Medicine & Hygiene; 80 (6):879-880. |
| Stephenson I, Wiselka M (2000) Drug treatment of tropical parasitic infections: Recent achievements and developments. Drugs; 60 (5):985-995. |
| Tagbor HK, Chandramohan D, Greenwood B. The safety of amodiaquine use in pregnant women. Expert Opin Drug Saf. 2007 Nov;6(6):631-5. Review. PubMed PMID: 17967151. |
| Tarning J, McGready R, Lindegardh N, Ashley EA, Pimanpanarak M, Kamanikom B, Annerberg A, Day NP, Stepniewska K, Singhasivanon P, White NJ, Nosten F. Population pharmacokinetics of lumefantrine in pregnant women treated with artemether-lumefantrine for uncomplicated Plasmodium falciparum malaria. Antimicrob Agents Chemother. 2009 Sep;53(9):3837-46. Epub 2009 Jun 29. PubMed PMID: 19564366; PubMed Central PMCID: PMC2737887. |
| Taylor TE (1994) Malaria: Epidemiology, pregnancy, and pediatric infections. Current Opinion in Infectious Diseases; 7 (5):536-541. |
| Taylor WR, Canon V, White NJ (2006) Pulmonary manifestations of malaria : recognition and management. Treatments in Respiratory Medicine; 5 (6):419-428. |
| Taylor WR, White NJ. Antimalarial drug toxicity: a review. Drug Saf. 2004;27(1):25-61. Review. PubMed PMID: 14720085. |
| Taylor WRJ, Canon V, White NJ (2006) Pulmonary manifestations of malaria: Recognition and management. Treatments in Respiratory Medicine; 5 (6):419-428. |
| Taylor WRJ, White NJ (2002) Malaria and the lung. Clinics in Chest Medicine; 23 (2):457-468. |
| Teklehaimanot HD, Teklehaimanot A, Kiszewski A, Rampao HS, Sachs JD. Malaria in São Tomé and principe: on the brink of elimination after three years of effective antimalarial measures. Am J Trop Med Hyg. 2009 Jan;80(1):133-40. PubMed PMID: 19141851. |
| Theiler RN. Evidence-based antimicrobial therapy in pregnancy: long overdue. Clinical Pharmacology & Therapeutics 2009;86(3):237-238 |
| Thomson E, Bannister B (2002) Prevention of malaria in travellers - A summary of current guidelines. CPD Infection; 3 (3):80-84. |
| Traore B, Muanza K, Looareesuwan S, et al (2000) Cytoadherence characteristics of Plasmodium falciparum isolates in Thailand using an in vitro human lung endothelial cells model. American Journal of Tropical Medicine and Hygiene; 62 (1):38-44. |
| Treeprasertsuk S, Silachamroon U, Krudsood S, et al (2010) Ursodeoxycholic acid and artesunate in the treatment of severe falciparum malaria patients with jaundice. Journal of Gastroenterology & Hepatology; 25 (2):362-368. |
| Trimnell AR, Kraemer SM, Mukherjee S, Phippard DJ, Janes JH, Flamoe E, Su XZ, Awadalla P, Smith JD. Global genetic diversity and evolution of var genes associated with placental and severe childhood malaria. Mol Biochem Parasitol. 2006 Aug;148(2):169-80. Epub 2006 Apr 18. PubMed PMID: 16697476. |
| Ugburo AO, Ilombu CA, Temiye EO, et al (2009) Severe idiosyncratic drug reaction (Lyells syndrome) after ingesting dihydroartemisinin. Nigerian journal of clinical practice; 12 (2):224-227 |
| Umar MT, Chika A, Jimoh AO (2011) Compliance of primary health care providers to recommendation of artemesinin-based combination therapy in the treatment of uncomplicated malaria in selected primary health care centres in Sokoto, North Western Nigeria. International Journal of Tropical Medicine; 6 (3):70-72. |
| Valecha N, Tripathi KD. Artemisinin: Current status in malaria. Indian Journal of Pharmacology 1997;43(6):71-75 |
| Vallely A, McCarthy J, Changalucha J, Vallely L, Chandramohan. Treating malaria in pregnancy in developing countries: priorities in clinical research and drug development. Expert Review of Clinical Pharmacology 2008;1(1);61-72 |
| Vallely A, Vallely L, Changalucha J, Greenwood B, Chandramohan D. Intermittent preventive treatment for malaria in pregnancy in Africa: What's new, what's needed? Malaria Journal 2007;6:16 |
| van Eijk AM, Terlouw DJ (2011) Azithromycin for treating uncomplicated malaria Systematic Review. Cochrane Database of Systematic Reviews; 5: |
| Van Geertruyden JP, Van EE, Yosaatmadja F, et al (2009) The relationship of Plasmodium falciparum humeral immunity with HIV-1 immunosuppression and treatment efficacy in Zambia. Malaria Journal; 8 (1): |
| Van VM, Van BA, Sicuri E, et al (2011) Malaria treatment and prophylaxis in endemic and nonendemic countries: Evidence on strategies and their cost-effectiveness. Future Microbiology; 6 (12):1485-1500. |
| Vashishtha VM (2008) World malaria report 2008: A billion-dollar moment for a centuries old disease? Indian Pediatrics; 45 (12):985-986. |
| Walker NF, Nadjm B, Whitty CJM (2010) Malaria. Medicine; 38 (1):41-46. |
| Walther B, Walther M (2007) What does it take to control malaria? Annals of Tropical Medicine and Parasitology; 101 (8):657-672. |
| Wang TY. Follow-up observation on the therapeutic effects and remote reactions of artemisinin (Qinghaosu) and artemether in treating malaria in pregnant woman. J Tradit Chin Med. 1989 Mar;9(1):28-30. PubMed PMID: 2761279. |
| Ward SA, Sevene EJP, Hastings IM, Nosten F. Antimalarial drugs and pregnancy: safety, pharmacokinetics, and pharmacovigilance. The Lancet Infectious Diseases 2007;11:136-144 |
| Warrell DA (1989) Treatment of severe malaria. Review 54 refs. Journal of the Royal Society of Medicine; 82 Suppl 17:44-50. |
| Warrell DA. Cerebral malaria: clinical features, pathophysiology and treatment. Ann Trop Med Parasitol. 1997 Oct;91(7):875-84. Review. PubMed PMID: 9625945. |
| Warsame M, Kimbute O, Machinda Z, et al (2007) Recognition, perceptions and treatment practices for severe malaria in rural Tanzania: implications for accessing rectal artesunate as a pre-referral. PLoS ONE; 2 (1):e149. |
| Warsame M, Olumese P, Mendis K. Role of medicines in malaria control and elimination. Drug Development Research 2010;1:4-11 |
| Warsame M, Olumese P, Mendis K. Role of medicines in malaria control and elimination. Drug Development Research 2010;1:4-11 |
| White NJ (1985) Clinical pharmacokinetics of antimalarial drugs. Review 133 refs. Clinical Pharmacokinetics; 10 (3):187-215. |
| White NJ (2004) Antimalarial drug resistance. Review 60 refs. Journal of Clinical Investigation; 113 (8):1084-1092. |
| White NJ (2005) Intermittent presumptive treatment for malaria: A better understanding of the pharmacodynamics will guide more rational policymaking. PLos Medicine; 2 (1):0028-0033. |
| White NJ, McGready RM, Nosten FH. New medicines for tropical diseases in pregnancy: catch-22. PLoS Med. 2008 Jun 17;5(6):e133. Review. PubMed PMID: 18563964; PubMed Central PMCID: PMC2429948. |
| White NJ, Pongtavornpinyo W, Maude RJ, et al (2009) Hyperparasitaemia and low dosing are an important source of anti-malarial drug resistance. Malaria Journal; 8 (1): 253 |
| White NJ, Pukrittayakamee S. Clinical malaria in the tropics. Med J Aust. 1993 Aug 2;159(3):197-203. Review. Erratum in: Med J Aust 1994 Sep 5;161(5):344. PubMed PMID: 8336622. |
| White NJ. The treatment of malaria. N Engl J Med. 1996 Sep 12;335(11):800-6. Review. PubMed PMID: 8703186. |
| White TE, Bushdid PB, Ritter S, Laffan SB, Clark RL. Artesunate-induced depletion of embryonic erythroblasts precedes embryolethality and teratogenicity in vivo. Birth Defects Res B Dev Reprod Toxicol. 2006 Oct;77(5):413-29. PubMed PMID: 17066416. |
| White TEK, Clark RL (2008) Sensitive periods for developmental toxicity of orally administered artesunate in the rat. Birth Defects Research Part B - Developmental and Reproductive Toxicology; 83 (4):407-417. |
| Whitty CJM, Edmonds S, Mutabingwa TK. Review. Malaria in pregnancy. BJOG 2005;112(9);1189-1195 |
| Whitty CJM, Sanderson F. New therapies and changing patterns of treatment for malaria. Curr Opin Infect Diseases 1999: 6;579-584 PM ID 17035824 |
| Wilby KJ, Ensom MH. Pharmacokinetics of antimalarials in pregnancy: a systematic review. Clin Pharmacokinet. 2011 Nov 1;50(11):705-23. doi: 10.2165/11594550-000000000-00000. PubMed PMID: 21973268. |
| Wilby KJ, Ensom MHH (2011) Erratum: Pharmacokinetics of antimalarials in pregnancy: A systematic review (Clin Pharmacokinet (2011) 50: 11 (705-723)). Clinical Pharmacokinetics; 50 (12):827. |
| Wilcox M, Falquet J, Ferreira JFS, et al (2007) Artemisia annua as a herbal tea for malaria. African Journal of Traditional, Complementary and Alternative Medicines; 4 (1):121-123. |
| Willcox ML, Burton S, Oyweka R, Namyalo R, Challand S, Lindsey K. Evaluation and pharmacovigilance of projects promoting cultivation and local use of Artemisia annua for malaria. Malar J. 2011 Apr 11;10:84. PubMed PMID: 21481234; PubMed Central PMCID: PMC3098208. |
| Willcox ML, Graz B, Diakite C, Falquet J, Dackouo F, Sidibe O, Giani S, Diallo D. Is parasite clearance clinically important after malaria treatment in a high transmission area? A 3-month follow-up of home-based management with herbal medicine or ACT. Trans R Soc Trop Med Hyg. 2011 Jan;105(1):23-31. Epub 2010 Nov 5. PubMed PMID: 21056445. |
| Willcox,M.L., Burton,S., Oyweka,R., Namyalo,R., Challand,S., Lindsey,K. Evaluation and pharmacovigilance of projects promoting cultivation and local use of Artemisia annua for malaria. Malaria Journal 2011. PubMed ID 21481234 |
| Willcox,M.L., Graz,B., Diakite,C., Falquet,J., Dackouo,F., Sidibe,O., Giani,S., Diallo,D. Is parasite clearance clinically important after malaria treatment in a high transmission area? A 3-month follow-up of home-based management with herbal medicine or ACT. Transactions of the Royal Society of Tropical Medicine and Hygiene 2011;1:23-31. PM ID 21056445 |
| Winstanley P (1998) Malaria: Treatment. Journal of the Royal College of Physicians of London; 32 (3):203-207. |
| Winstanley P (2003) The contribution of clinical pharmacology to antimalarial drug discovery and development. British Journal of Clinical Pharmacology; 55 (5):464-468. |
| Winstanley P, Ward S (2006) Malaria Chemotherapy. Advances in Parasitology; 61:47-76. |
| Worrall E, Morel C, Yeung S, et al (2007) The economics of malaria in pregnancy-a review of the evidence and research priorities. Lancet Infectious Diseases; 7 (2):156-168. |
| Xu JH, Zhang YP. Contragestational effects of dihydroartemisinin and artesunate. Yao Xue Xue Bao 1996; 31(9);657-661. PubMed ID 9863230 |
| Yakoob MY, Lawn JE, Darmstadt GL, et al (2010) Stillbirths: Epidemiology, Evidence, and Priorities for Action. Seminars in Perinatology; 34 (6):387-394. |
