# Supplementary material for: A systematic review of the safety and efficacy of artemether-lumefantrine against uncomplicated Plasmodium falciparum malaria during pregnancy
Source: Malar J. 2012 May 1;11:141. doi: 10.1186/1475-2875-11-141 (PMC3405476; doi:10.1186/1475-2875-11-141)
Supplement: Additional file 4 — Publications reporting on the safety, efficacy and pharmacokinetics of artemether and artemether-lumefantrine in pregnancy, including the number of exposures during pregnancy. Sixteen articles were identified by the literature search, all reporting on pregnancies exposed to artemether or artemether-lumefantrine. The number of pregnancies exposed to artemether or artemether-lumefantrine are reported, and it is noted where exposures have been previously reported. AL, artemether-lumefantrine; AE, adverse events; SAE, serious adverse events; SP, sulphadoxine-pyrimethamine; ACT, artemisinin-based combination therapies; †National treatment policy is quinine throughout pregnancy [55-57]; †National treatment policy is quinine in the first trimester and ACT in the second and third trimesters of pregnancy [55,58,59]; §National treatment policy is quinine in the first trimester and SP in the second and third trimesters of pregnancy; Benin,† Burkina Faso,† Cameroon,† DRC,† Gabon,† Mozambique,† Mali,† Thailand,† Ghana,‡ Kenya,‡ Malawi,‡ Nigeria,‡ Sudan,‡ Tanzania,‡ Uganda,‡ Zambia,§[26,55,57-59]; #McGready et al., 2001 [17] includes all of the artemether exposures reported across three publications. To avoid duplication of pregnancy exposures the other two reports have not been included [18,19]; ¶Included women with a pregnancy in the last 12 months that lasted until at least the third trimester. Current pregnancies were excluded; ††Mali,† Mozambique,† Thailand,† Kenya,‡ Nigeria,‡ Sudan,‡ Tanzania,‡ Zambia§[26,55,57,59]; ‡‡National treatment policy is quinine/AL [60]. Wang, 1989 [20], Sowunmi et al., 1998 [15], McGready et al., 2001 [17], Adam et al., 2004 [16], McGready et al., 2006 [21], Dellicour et al., 2007 [14], McGready et al., 2008 [22], Kaye et al., 2008 [23], Orton et al., 2008 [61], Adam et al., 2009 [24], Tarning et al., 2009 [37], Piola et al., 2010 [25], Manyando et al., 2010 [26], McGready et al., 2011 [29], Sangaré et al., 2011 [27], Wilby and Ensom, 2011 [30]. [file 1475-2875-11-141-S4.doc]

| **Publication** | **Description** | **Location, year** | **Number of pregnancies exposed to artemether or artemether-lumefantrine** | **Safety data** | **Efficacy and pharmacokinetic data** |
| --- | --- | --- | --- | --- | --- |
| **Wang, 1989 [20]** | Observational, artemisinin and artemether, 2^nd^–3^rd^ trimester | China^†^, 1976–1980 | 2 exposed to artemether in the 2^nd^–3^rd^ trimester | Condition of labour, growth and development (5–9 years), congenital malformation | Parasite and fever clearance (time), treatment failure (Day 28) |
| **Sowunmi *et al,* 1998 [15]** | Randomized, artemether *vs* artemether + mefloquine, 2^nd^–3^rd^ trimester | Nigeria,^‡^ 1994–1997 | 45 exposed to artemether in the 2^nd^–3^rd^ trimester | Maternal AE, physical and neuro-developmental assessment of newborns, infant neuro-development (6–36 months) | Parasite and fever clearance (time) |
| **McGready *et al*, 2001 [17]** | Observational study, artesunate and artemether^#^ | Thailand,^†^ 1992–2000 | 10 exposed to artemether, 1 exposed to artemether-lumefantrine. Timing of exposures was not reported | AE, prematurity, miscarriage, stillbirth, birth weight | Treatment failure (Day 42) |
| **Adam *et al,* 2004 [16]** | Observational study, artemether, 1^st^–3^rd^ trimester | Sudan,^‡^ 1997–2001 | 27 exposed to artemether in the 2^nd^–3^rd^ trimester, 1 exposed to artemether in the 1^st^ trimester | Maternal death, stillbirth, miscarriage, congenital abnormality, infant death | Treatment failure (Day 28) |
| **McGready *et al,* 2006 [21]** | Pharmacokinetic study, AL, 2^nd^–3^rd^ trimester | Thailand,^†^ 2004 | These exposures were also reported in McGready *et al*, 2008 | Maternal AE, prematurity, congenital abnormality, birth weight, infant development (1 year) | Parasite clearance (time), plasma level (artemether, lumefantrine) |
| **Dellicour *et al,* 2007 [14]** | Retrospective review, 14 studies, artemether and artesunate, 1^st^–3^rd^ trimester (includes Wang 1989, Sowunmi *et al*, 1998, McGready *et al*, 2001, Adam *et al*, 2004) | Gambia,^†^ Thailand,^†^ China,^†^ Nigeria,^‡^ Sudan,^‡^ 1976–2005 | These exposures were also reported in Wang 1989, Sowunmi *et al*, 1998, McGready *et al*, 2001, Adam *et al*, 2004 | Maternal AE, miscarriage, stillbirth, prematurity, neonatal death, congenital abnormality, birth weight, developmental delay |  |
| **McGready *et al*, 2008 [22]** | Open-label, randomized study, AL *vs.* artesunate, 2^nd^–3^rd^ trimester | Thailand,^†^ 2004–2006 | 125 exposed to artemether-lumefantrine in the 2^nd^–3^rd^ trimester | Maternal AE, prematurity, birth weight, congenital abnormality, neurological development, infant development (1 year) | Parasite and fever clearance (time), cure rate (delivery or Day 42 if later), parasite positive at delivery (maternal, cord, placental blood) |
| **Kaye *et al*, 2008 [23]** | Open-label, randomized study, AL *vs.* chlorproguanil-dapsone, 2^nd^–3^rd^ trimester | Uganda,^‡^ 2006 | 58 exposed to artemether-lumefantrine in the 2^nd^–3^rd^ trimester | Maternal AE | Parasite and fever clearance (time), treatment failure (Day 28) |
| **Orton *et al*, 2008 [61]** | Cochrane review, 10 studies, anti-malarials, uncomplicated malaria (includes Sowunmi *et al*, 1998) | Burkina Faso,^†^ DRC,^†^ Ghana,^‡^ Malawi,^‡^ Nigeria,^‡^ Thailand,^†^ 1993–2004 | These exposures were also reported in Sowunmi *et al*, 1998 | Maternal AE, SAE, AE resulting in discontinuation, miscarriage, still birth, prematurity, birth weight, perinatal death, neonatal malaria, congenital abnormality, congenital anaemia or neonatal haemoglobin | Treatment failure (Day 28 or 42), fever and parasite clearance (time), anaemia |
| **Adam *et al,* 2009 [24]** | Observational study, artemisinins, 1^st^ trimester | Sudan,^‡^ 2006–2008 | 48 exposed to artemether in the 1^st^ trimester, 3 exposed to artemether-lumefantrine in the 1^st^ trimester | Maternal death, miscarriage, prematurity, congenital abnormality, infant death |  |
| **Tarning *et al,* 2009 [37]** | Pharmacokinetic study, AL, 2^nd^–3^rd^ trimester | Thailand,^†^ 2004–2006 | These exposures were also reported in McGready *et al*, 2008 |  | Plasma level (lumefantrine) |
| **Piola *et al,* 2010 [25]** | Open-label, randomized study, AL *vs.* quinine, 2^nd^–3^rd^ trimester | Uganda,^‡^ 2006–2009 | 152 exposed to artemether-lumefantrine in the 2^nd^–3^rd^ trimester | Maternal AE, prematurity, congenital abnormality, birth weight, perinatal and neonatal death, infant death | Cure rate (delivery or Day 42 if later), plasma level (Day 7, lumefantrine) |
| **Manyando *et al,* 2010 [26]** | Observational study, AL *vs* SP, 1^st^–3^rd^ trimester | Zambia,^§^ 2004–2008 | 348 exposed to artemether-lumefantrine in the 2^nd^–3^rd^ trimester, 156 exposed to artemether-lumefantrine in the 1^st^ trimester | Maternal AE, maternal death, perinatal death, miscarriage, stillbirth, prematurity, birth weight, congenital abnormality, gestational age at delivery, neurodevelopment (14 weeks, 12 months) |  |
| **McGready *et al,* 2011 [29]** | Systematic review, 60 studies, anti-malarials, 1^st^–3^rd^ trimester (includes Sowunmi *et al*, 1998, McGready *et al*, 2001, Adam *et al*, 2004, McGready *et al*, 2006, McGready *et al*, 2008, Kaye *et al*, 2008, Tarning *et al*, 2009) | Africa and Asia,^║^1991–2009 | These exposures were also reported in Sowunmi *et al*, 1998, McGready *et al*, 2001, Adam *et al*, 2004, McGready *et al*, 2006, McGready *et al*, 2008, Kaye *et al*, 2008, Tarning *et al*, 2009 |  | Treatment failure, gametocyte carriage, placental malaria |
| **Sangaré *et al,* 2011 [27]** | Retrospective, population study, self reports of anti-malarial use for self-reported malaria episodes^¶^, 1^st^–3^rd^ trimester | Uganda^‡^, 2008–2009 | 207 exposed to artemether-lumefantrine in the 2^nd^–3^rd^ trimesters, 53 exposed to artemether-lumefantrine in the 1^st^ trimester | Pregnancy outcome |  |
| **Wilby and Ensom, 2011 [30]** | Systematic review, 20 studies, pharmacokinetics of anti-malarials, 1^st^–3^rd^ trimester (includes McGready *et al*, 2006, Tarning *et al*, 2009) | Africa, Asia,^††^ Papua New Guinea,^‡‡^ 1996–2008 | These exposures were also reported in McGready *et al*, 2006, Tarning *et al*, 2009 |  | Plasma levels (artemether, lumefantrine) |
